# Accurate fine-grained weed instance segmentation amidst dense crop canopies using CPD-WeedNet

**DOI:** 10.3389/fpls.2025.1632684

**Published:** 2025-09-03

**Authors:** Lan Luo, Jinfan Wei, Lingyun Ni, Cun Pei, Haotian Gong, Hang Zhu, Caocan Zhu, Mengchao Chen, Ye Mu, He Gong

**Affiliations:** ^1^ College of Information Technology, Jilin Agricultural University, Changchun, China; ^2^ Jilin Province Intelligent Environmental Engineering Research Center, Changchun, China

**Keywords:** precision agriculture, field weed segmentation, instance segmentation, fine-grained recognition, CPD-WeedNet

## Abstract

Precisely segmenting multi-category farmland weeds is of great significance for achieving targeted weeding and sustainable agriculture. However, the similar morphology between field crops and weeds, complex occlusions, variable lighting conditions, and the diversity of target scales pose severe challenges to the accuracy and efficiency of existing methods on resource-constrained platforms. This study proposes a novel instance segmentation framework, CPD-WeedNet, specifically designed for fine-grained weed identification in complex field scenarios. CPD-WeedNet innovatively presents three core components: the CSP-MUIB backbone module, which enhances the discriminative ability of initial features at a low computational cost; the PFA neck module, which efficiently integrates shallow-layer details to improve the contour capture of small and medium-sized targets; and the DFS neck module, which utilizes the Transformer to enhance global context understanding and cope with large targets and complex occlusions. On a self-constructed soybean field weed dataset, CPD-WeedNet achieved 80.6% mAP50(Mask) and 85.3% mAP50(Box), with pixel-level mIoU and mAcc reaching 86.6% and 94.6% respectively, significantly outperforming mainstream YOLO baselines. On the public Fine24 dataset, CPD-WeedNet attained 75.4% mIoU, 81.7% mAcc, and 65.9% mAP50 (Mask), demonstrating an excellent balance between performance and efficiency. The proposed CPD-WeedNet achieves an excellent balance between performance and efficiency, demonstrating its significant potential as a key vision technology for the development of low-cost, real-time intelligent weeding systems. This research is of great significance for promoting precision agriculture.

## 1 Introduction

In the realm of agricultural production, weed infestations are both prevalent and highly detrimental. Competing with crops for water, nutrients, living space, and sunlight, their strong adaptability poses a serious threat to the growth and development of cash crops, leading to a decline in crop yields and a deterioration in quality (Vilà et al., 2021; Montull and Torra, 2023). Moreover, weeds often serve as intermediate hosts for pests and diseases, increasing the risk of pathogen transmission and further endangering crop health and the final harvest (Özlüoymak, 2022).

Weed damage is generally inevitable in the process of agricultural production. Currently, the strategies for controlling farmland weeds mainly rely on three aspects: chemical (Damalas and Koutroubas, 2024), biological (Winston et al., 2024; Clay, 2021), and physical (Upadhyay et al., 2024) methods. Among them, chemical weeding is widely used due to its high efficiency. However, the long-term and large-scale use of herbicides not only causes increasingly serious environmental pollution problems but also induces changes in the population structure of farmland weeds. Gradually, tolerant weed populations evolve into dominant ones, significantly increasing the difficulty and cost of weed management (Baucom, 2019). The traditional non-selective, large-area spraying operation mode not only has low weed control efficiency and lacks pertinence but also exacerbates pesticide waste and environmental pollution. Furthermore, traditional non-selective, large-area spraying is inefficient and untargeted, exacerbating pesticide waste and environmental contamination. With the growing global concern over pesticide use and residue issues, as well as the enhanced awareness of a green environment and food safety, developing technologies capable of accurately identifying and locating weeds to achieve targeted spraying has become one of the key issues that urgently need to be addressed and the most effective approaches in this field (Ehrampoosh et al., 2025).

Computer vision provides a key solution for precise and automated weed identification in modern agriculture. Traditional methods, typically based on machine learning, rely on hand-crafted features such as color, shape, or texture to distinguish between crops and weeds (Adhinata et al., 2024; Rakhmatuiln et al., 2021). However, such approaches often lack robustness in complex field environments characterized by variable illumination and vegetation occlusion. With the advent of deep learning, researchers have explored various simplification strategies for rapid implementation. One such strategy is indirect identification, where, for instance, Osorio et al. first employed a deep learning model to accurately locate lettuce crops and subsequently classified all non-lettuce vegetation as “weeds” via logical exclusion (Osorio et al., 2020). In parallel, a more direct simplification is ternary semantic segmentation, which classifies image pixels into “soil,” “crop,” and “weed.” For this purpose, Moazzam et al. designed a sophisticated W-shaped network that utilizes a two-stage encoder-decoder structure to first separate vegetation from the background and then differentiate between crops and weeds, addressing their high visual similarity (Moazzam et al., 2023). While both strategies have achieved promising results, they share a common limitation: treating diverse weed populations as a single monolithic “weed” category, thereby failing to achieve species-level, fine-grained classification. This shortcoming hinders the implementation of more advanced Site-Specific Weed Management (SSWM) strategies that rely on the biological characteristics of weeds, such as their growth cycles or herbicide resistance.

The rapid development of deep learning, particularly Convolutional Neural Networks (CNNs), has led to significant breakthroughs in weed identification. Deep learning-based models can automatically learn high-level discriminative features from raw images, vastly outperforming traditional methods in both accuracy and robustness. This technological wave has given rise to two mainstream application pathways, centered on Unmanned Aerial Vehicles (UAVs) and ground-based mobile robots, each with a distinct operational focus. Leveraging the advantage of rapid, large-scale field coverage, the UAV-based approach is primarily used for mapping the geospatial distribution of weeds (Huang et al., 2018). The core challenge of this pathway is extracting fine-grained features sufficient to distinguish between morphologically similar species from limited-resolution aerial imagery. To this end, researchers have focused on constructing more advanced deep learning architectures. For example, to address the high morphological similarity between rice and weeds, Guo et al. proposed an innovative framework named CTFFNet (Guo et al., 2025). This framework utilizes a CNN to capture local texture details in parallel with a Transformer for global contextual information, ultimately fusing these features via a CBAM attention mechanism. Similarly, to address significant target scale variations and limited resolution in UAV imagery of soybean fields, Xu et al. employed an encoder-decoder architecture based on ResNet101_v, enhancing multi-scale feature extraction with depth-separable dilated spatial pyramid pooling (Xu et al., 2023). Despite excelling in semantic segmentation tasks, these approaches remain limited in their ability to accurately identify weeds heavily occluded by the crop canopy. In contrast, the ground-robot-based approach focuses on close-range, high-precision real-time identification and immediate removal operations (Li et al., 2022). Due to the shorter distance between the sensors and the targets, this approach has a natural advantage in dealing with occlusion problems. Consequently, the research focus has shifted to developing lightweight instance segmentation models that can meet the real-time processing demands of onboard edge-computing platforms. For instance, for resource-constrained laser weeding, Lyu et al. proposed an improved BFFDC-YOLOv8-seg model (Lyu et al., 2024). It enhances small-target detection by incorporating a Bidirectional Feature Pyramid Network (BiFPN) and employs Dynamic Snake-shaped Convolution to delineate weed stem contours, achieving a model size of just 6.8MB at 24.8 FPS. In the complex environment of tea plantations, Cao et al. improved the distinction between similarly colored weeds and tea plants by integrating a CBAM attention mechanism and Atrous Spatial Pyramid Pooling (ASPP) into a U-Net architecture (Cao et al., 2025). These ground-based applications demonstrate that algorithm design must balance accuracy and efficiency. In summary, whether it is aerial mapping or ground-based precise removal, deep learning algorithms have become the core driving force. At the same time, this also reveals a common challenge that permeates both technological approaches: how to achieve precise, robust, and efficient weed identification under the unique constraints of different operating platforms.

Building on this common challenge, the research focus has shifted from simple “crop-weed” binary identification to the more demanding task of multi-species, fine-grained weed recognition. This transition places higher demands on the discriminative power of algorithms, as many weed species are morphologically very similar. An important research direction is to classify weeds based on their biological characteristics to guide precision management. For instance, Torres-Sánchez et al. successfully distinguished between broad-leaved and grass weeds using UAV imagery, providing a foundation for selective herbicide application (Torres-Sánchez et al., 2021). Taking this principle a step further, Jin et al. classified weeds directly by their susceptibility to herbicides with different modes of action (e.g., ACCase inhibitors), enabling a more advanced level of precision spraying (Jin et al., 2025). However, achieving such fine-grained classification is exceptionally challenging, as it requires the model to capture subtle visual differences between species.

In parallel, another research frontier aimed at improving practical applicability is reducing the reliance on large-scale, meticulously annotated data. This dependency represents a major bottleneck for the real-world application of deep learning models. Semi-supervised learning (SSL) leverages a small set of labeled data to guide a model’s learning from a large corpus of unlabeled data. For instance, Saleh et al. constructed a “teacher-student” mutual learning framework where a more stable “teacher” model generates “pseudo-labels” for unlabeled images (Saleh et al., 2025). These pseudo-labels are then used alongside the ground-truth labels to train the “student” model, thereby significantly amplifying the value of the limited annotated data. Unsupervised Domain Adaptation (UDA), in contrast, focuses on the generalization problem from a source domain to a new target domain without requiring any labels from the target. For example, Ilyas et al. employed adversarial training to compel their model to learn domain-invariant features—essential characteristics of weeds that remain constant across different farms—while ignoring domain-specific artifacts caused by variations in soil type and lighting conditions (Ilyas et al., 2023). Although both methods are highly promising and can significantly lower the threshold of data preparation, they also face their respective challenges. For example, in SSL, ensuring the quality of pseudo-labels, and in UDA, effectively aligning the feature distributions of different domains are still key technical problems to be solved in this field. Synthesizing the preceding analysis, a core research gap becomes evident: approaches pursuing high-precision, fine-grained recognition typically rely on complex, computationally intensive architectures, while methods prioritizing efficiency and a low-entry barrier are still evolving and face their own unique challenges. Therefore, the field urgently requires a solution that strikes an optimal balance between a lightweight architecture with high inference efficiency and the capability for precise instance segmentation of multi-class, morphologically similar weeds. This need is especially acute for potential deployment on resource-constrained platforms like UAVs and ground robots, where existing high-performance models are often too cumbersome, while traditional lightweight models fail to meet the fine-grained recognition accuracy demanded by precision agriculture.

To address this critical challenge, this study presents a novel lightweight instance segmentation framework optimized for precision agriculture, CPD-WeedNet. This framework aims to resolve the aforementioned contradiction between model performance and computational efficiency through its elaborate architectural design. CPD-WeedNet achieves this goal by integrating three core modules that work in concert: an efficient CSP-MUIB backbone network, which is designed to extract more discriminative basic features at a low computational cost; and the PFA and DFS modules that collaborate within the neck network. The PFA module focuses on efficiently fusing shallow-layer details to enhance the accuracy of contour capture for small - and medium-sized targets. Meanwhile, the DFS module utilizes the Transformer to strengthen the global context understanding ability, thereby dealing with large or severely occluded targets. This design enables CPD-WeedNet to maintain a lightweight model while enabling accurate and robust instance segmentation of multi-category, highly similar weeds.

The main contributions of this study can be summarized as follows:

1. We propose a novel instance segmentation network, CPD-WeedNet, specifically optimized for agricultural scenarios. Its architecture is designed to tackle challenges such as the high morphological similarity between crops and multiple weed species, variable growth forms, and interference from complex field backgrounds.
2. We designed and integrated a novel and efficient feature extraction module, namely CSP-MUIB. This module significantly enhances the model’s feature representation capabilities while maintaining a low computational cost, providing superior feature support for subsequent tasks.
3. We propose two innovative multi-scale feature fusion modules for the neck network: PFA and DFS. PFA enhances detail features via asymmetric convolutions and a progressive aggregation mechanism, while DFS innovatively combines CNNs with a Transformer to jointly inject richer, higher-quality multi-scale semantic information into the segmentation task.
4. We constructed an instance segmentation dataset comprising soybeans, poaceous weeds, and broadleaf weeds. Through comprehensive experiments on both our self-constructed dataset and the public Fine24 dataset, we verify that our proposed model achieves an excellent balance between accuracy, efficiency, and generalization.

## 2 Methods and materials

**Figure 1:**
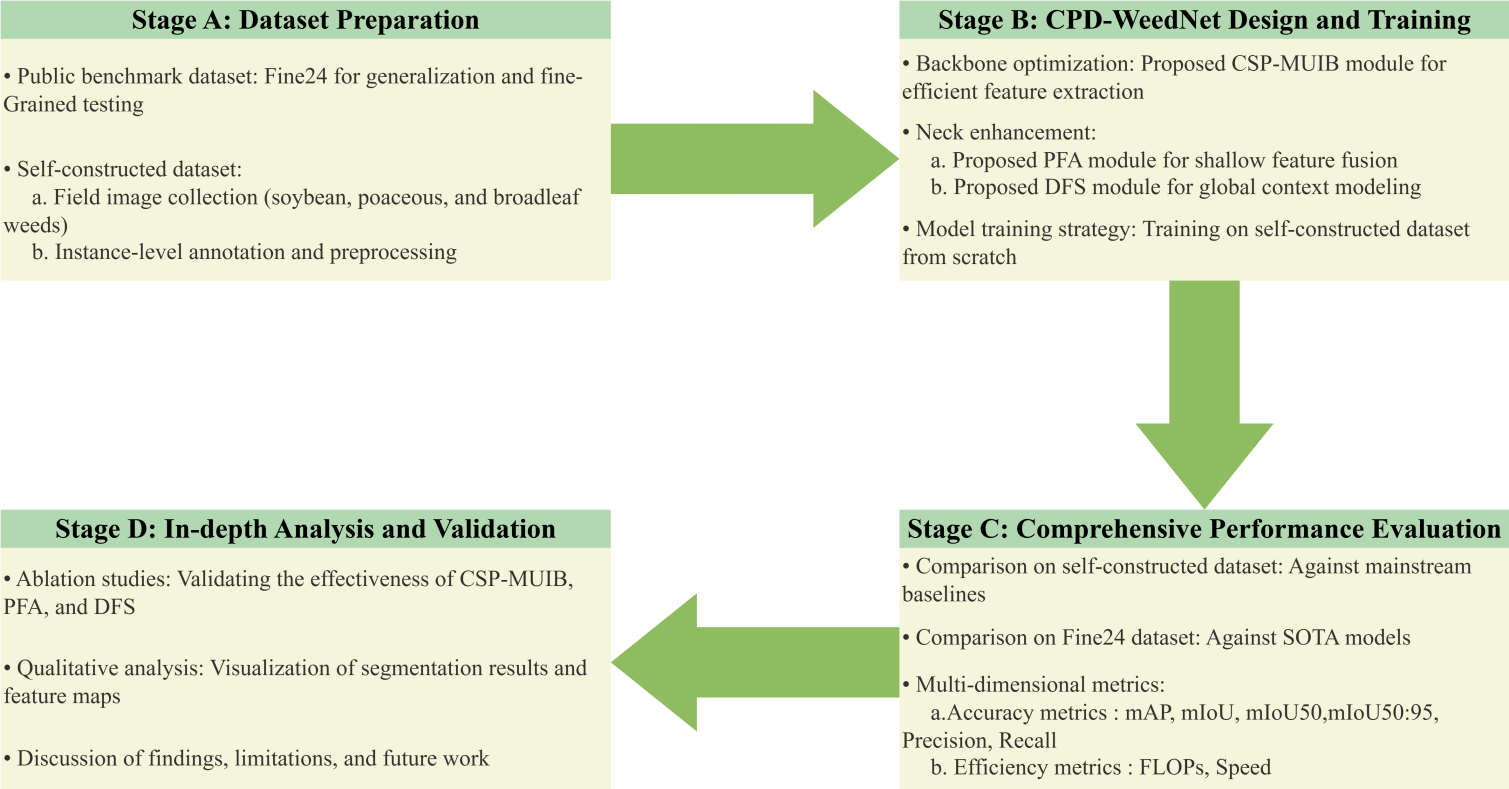
The overall workflow of this study. The research is structured into four core stages: **(A)** Dataset Preparation, involving the construction of a self-constructed dataset and the adoption of a public benchmark; **(B)** CPD-WeedNet Design and Training, focusing on the proposed novel architecture; **(C)** Comprehensive Performance Evaluation, benchmarking our model against baseline and state-of-the-art methods; and **(D)** In-depth Analysis and Validation, which includes ablation studies and qualitative assessments.

The overall workflow of this study is illustrated in **Figure 1**, comprising four core stages. This section details the first two stages: dataset construction (Section 2.1) and the design of the CPD-WeedNet model (Section 2.2). The subsequent sections will present the comprehensive performance evaluation (Section 3), an in-depth analysis and discussion (Section 4), and a final summary of the study (Section 5).

### 2.1 Experimental datasets

#### 2.1.1 Self-constructed dataset

**Figure 2:**
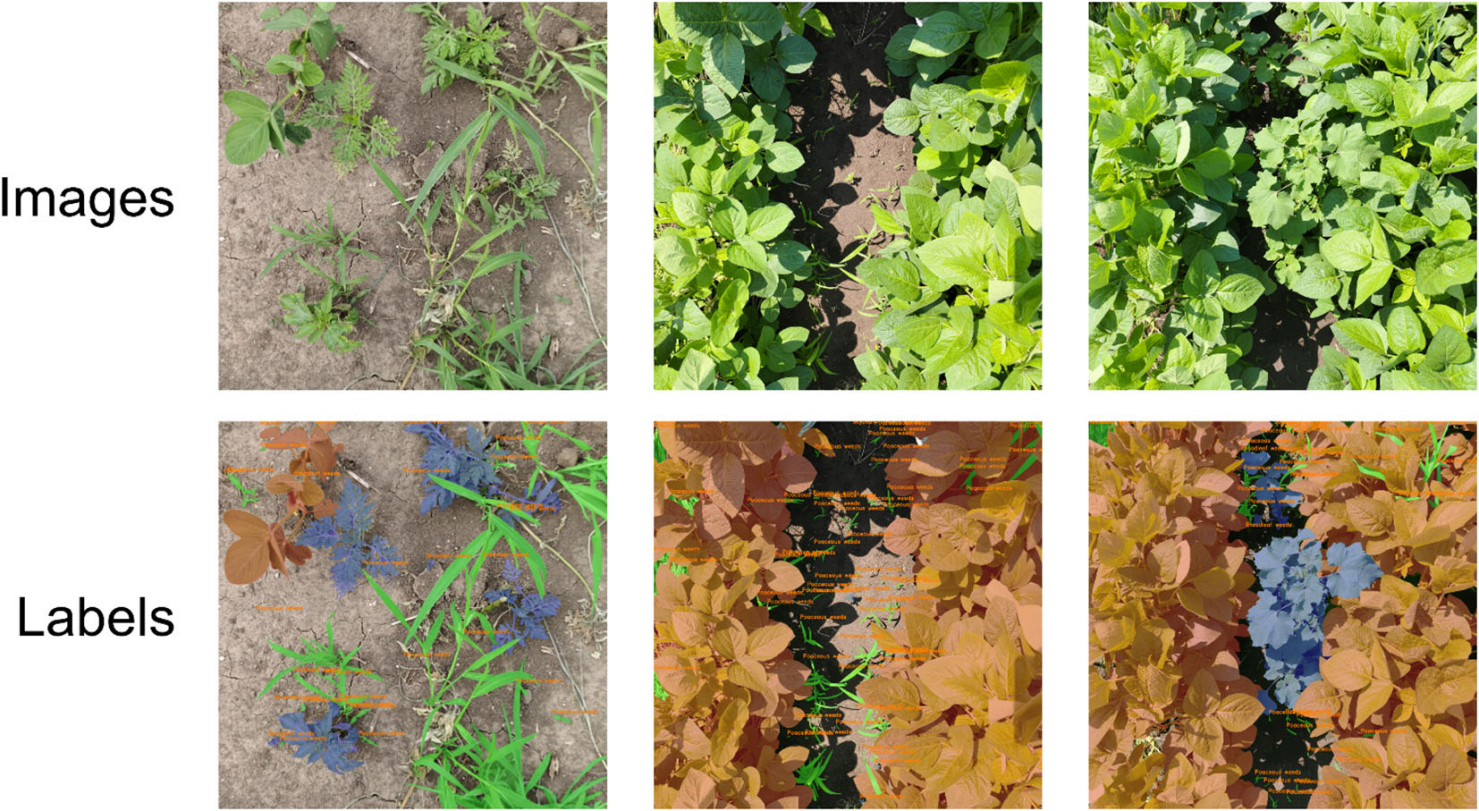
Some examples of the self-constructed dataset.

The self-constructed dataset utilized in this study was collected from multiple soybean experimental fields at Jilin Agricultural University between June 30 and August 16, 2024, using a smartphone. The dataset is designed to comprehensively simulate the complexity of real-world field environments and includes three target categories: soybeans, poaceous weeds, and broadleaf weeds. To ensure dataset diversity, our collection covered a broad spectrum of natural lighting conditions, ranging from the harsh shadows and specular highlights on leaves caused by direct midday sun to the soft, diffused light of overcast days. Furthermore, the dataset captures critical phenological variations, encompassing the key vegetative and early reproductive growth stages of soybeans (from V3, third trifoliolate, to R1, beginning bloom) and the entire developmental trajectory of weeds from seedling to maturity, thereby introducing significant target scale variability. The dataset is also rich with complex visual scenarios, featuring intricate occlusion patterns such as weeds obscured by the crop canopy, crops partially hidden by weeds, and multiple weed species growing densely intertwined. To establish the foundation for our study, we selected and meticulously annotated 600 original images at a resolution of 3060×3060 pixels, with representative examples shown in **Figure 2**. Based on the statistics of the normalized bounding box sizes, the proportions of large, medium, small, and tiny targets in the dataset are 20.73%, 35.66%, 25.07%, and 18.53% respectively. Subsequently, this dataset is divided into a training set, a validation set, and a test set at a ratio of 8:1:1. To prevent overfitting during the model training process, we applied data augmentation techniques to the training set and the validation set. The specific methods include random rotation of ±30°, addition of random noise, random translation, random vertical flip, and random adjustment of image brightness. The changes in the number of instances in each subset before and after data augmentation are detailed in **Table 1**.

**Table 1:** Instance details of the self-constructed dataset.

Classes	Train		Val		Test	Total
	Before	After	Before	After		
Soybean	748	4488	93	558	94	5981
Poaceous weeds	4190	25140	524	3144	524	62923
Broadleaf weeds	570	3420	71	426	72	4559
Total	5508	33048	688	4128	690	73463

#### 2.1.2 Public dataset

**Figure 3:**
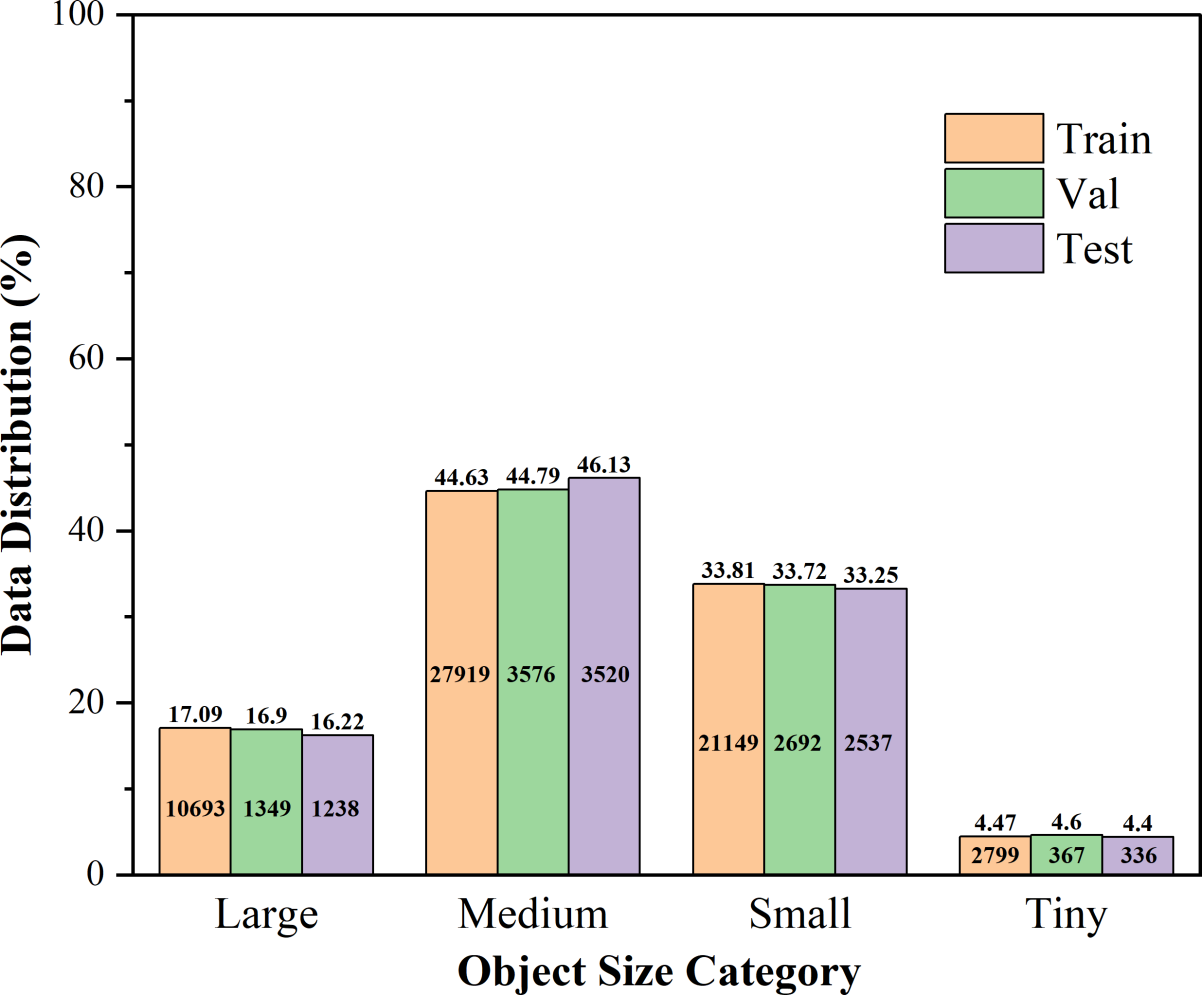
Distribution of instances in the Fine24 dataset by Normalized Size. In the figure, instances are classified according to the area of their bounding boxes in images with a resolution of 1920x1088. The specific definitions are as follows: Large (> $128^{2}$ ), Medium ( $32^{2}$ < area ≤ $128^{2}$ ), Small ( $16^{2}$ < area ≤ $32^{2}$ ), Tiny (area $\leq 16^{2}$ ).

To evaluate the generalization performance of our proposed model in large-scale, multi-category segmentation scenarios, we conducted experiments on the Fine24 dataset. As the most fine-grained and comprehensive variant of the CropAndWeed dataset suite (Steininger et al., 2023), Fine24 classifies plants into 24 categories, comprising 8 crop and 16 weed types. We partitioned the dataset into 6,135 training, 766 validation, and 768 test images. As illustrated in **Figure 3**, medium- and small-sized instances constitute the majority of the dataset, collectively accounting for nearly 80% of all instances. This distribution requires the model to possess excellent detail-capturing capabilities and a strong ability to discriminate between small- to medium-sized targets. Conversely, while tiny instances are less frequent, their inherent detection difficulty and relative scarcity could pose a learning bottleneck. Meanwhile, large instances, making up approximately 17% of the data, are crucial for assessing whether the model might overemphasize smaller targets at the expense of larger ones, thereby testing its capacity to balance learning across different scales.

**Table 2** details the distribution of instances in the Fine24 dataset. It conducts a hierarchical statistics of the training set and the validation set according to 24 fine-grained plant categories and four instance sizes. These data provide precise numerical evidence for the distribution characteristics shown in **Figure 3**. The dataset contains a total of 78,175 annotated instances, with 62,560 for training, 7,984 for validation, and 7,631 for the test set.

**Table 2:** The instance distribution quantified by category and size in the Fine24 dataset, including the specific quantities of instances of different categories and sizes in the training set and validation set.

Classes	Train	Val	Test	Total
Maize	2226	577	584	3387
Sugar beet	4830	660	539	6029
Soy	2669	410	297	3376
Sunflower	1564	161	167	1892
Potato	257	57	29	343
Pea	510	51	93	654
Bean	794	127	132	1053
Pumpkin	463	76	66	605
Grasses	16906	2048	1912	20866
Amaranth	2681	369	336	3386
Goosefoot	2808	308	402	3518
Knotweed	2457	232	356	3045
Corn spurry	685	100	156	941
Chickweed	170	41	17	228
Solanales	823	80	114	1017
Potato weed	2096	252	268	2616
Chamomile	2253	315	274	2842
Thistle	6980	819	801	8600
Mercuries	906	144	116	1166
Geranium	4040	703	450	5193
Crucifer	1464	190	221	1875
Poppy	736	53	101	890
Plantago	1015	135	156	1306
Labiate	531	76	44	651
Total	62560	7984	7631	78175

### 2.2 CPD-WeedNet segmentation network

In the field of lightweight instance segmentation, the YOLO (You Only Look Once) series of models has become one of the mainstream methods due to their excellent computational efficiency and relatively small number of parameters. This makes them particularly suitable for deployment on resource-constrained agricultural automation platforms to perform real-time and efficient field visual perception tasks. It is worth noting that there are significant differences in the evaluation paradigm between the YOLO series of instance segmentation models and traditional semantic segmentation models. This difference is rooted in the dual objectives of the instance segmentation task. It not only requires accurate pixel-level classification, but more importantly, it demands precise localization, category identification of each individual target instance, and meticulous delineation of its boundary contours. Given the great potential and continuous development of the YOLO framework in object detection and instance segmentation tasks, we developed and optimized the model based on the widely used Ultralytics codebase, and selected YOLOv11-seg as the basic architecture. To balance model accuracy and computational resource consumption, we set both the model width and depth scaling factors to 0.5. YOLOv11-seg is itself built upon the YOLOv8 architecture, with its main improvement lying in the backbone network: the original C2F module is replaced by the C3K2 module. Essentially, the C3K2 module is a flexible variant of C2F. Through internal parameter control, it can switch between two configurations: the C2F configuration and the configuration where the internal Bottleneck is replaced by the C3 module. Additionally, YOLOv11-seg introduces the C2PSA module after the SPPF module. This module enhances feature extraction and the attention mechanism by integrating the PSA (Pointwise Spatial Attention) block, improving the model’s sensitivity to key features. Its detection head draws on the design of YOLOv10, adopting depth-separable convolutions to reduce computational redundancy.

**Figure 4:**
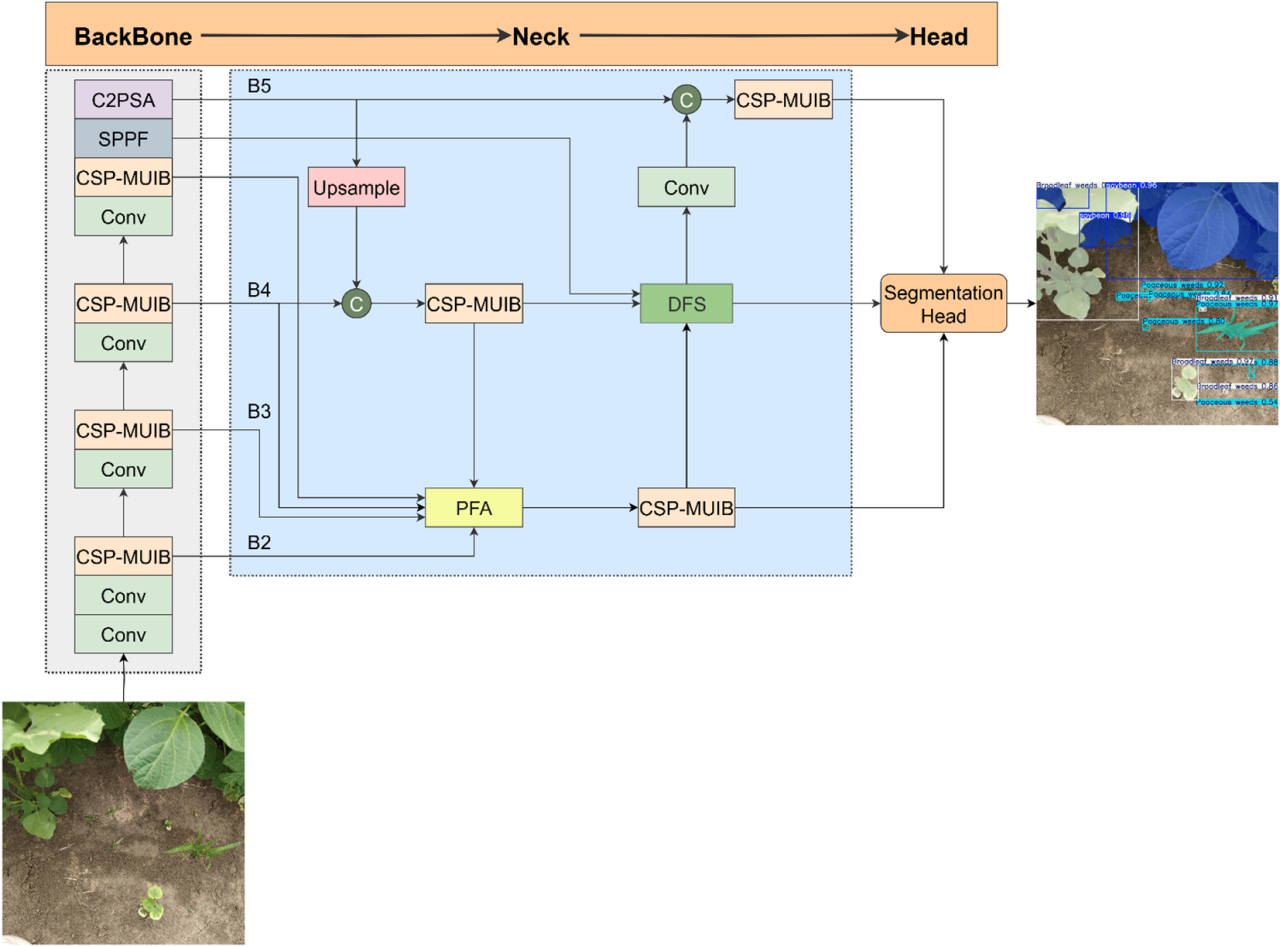
The overall network structure of CPD-WeedNet.

Although YOLOv11-seg has the above-mentioned advantages, to further enhance its ability to accurately identify crops and weeds in complex agricultural scenarios, especially in the face of challenges such as large variations in target size and diverse backgrounds, we have carried out targeted optimizations on its backbone network and neck network. The core objective is to enhance the model’s ability to capture complex relationships among features, effectively integrate contextual semantic information, and improve the representation performance for multi-scale targets. To this end, we propose the CPD-WeedNet model, and the overall structure is shown in **Figure 4**. In this model, we replace the feature extraction module C3K2 in the original backbone network with the newly proposed CSP-MUIB module, aiming to utilize its efficient and powerful feature representation ability to provide better basic features. At the same time, we introduce two innovative feature fusion modules proposed by us, the Progressive Feature Aggregator (PFA) and the Deep Feature Synthesizer (DFS), into the feature fusion path of the neck network. These two modules are designed to perform more refined and powerful multi-scale feature fusion: PFA focuses on efficiently aggregating multi-level features and enhancing detailed information, while DFS combines the Transformer mechanism to capture global dependency relationships. Together, they inject richer and higher-quality semantic information into different levels of the feature pyramid, ultimately serving the more accurate segmentation task.

#### 2.2.1 Cross-stage pooling unified inverted block

**Figure 5:**
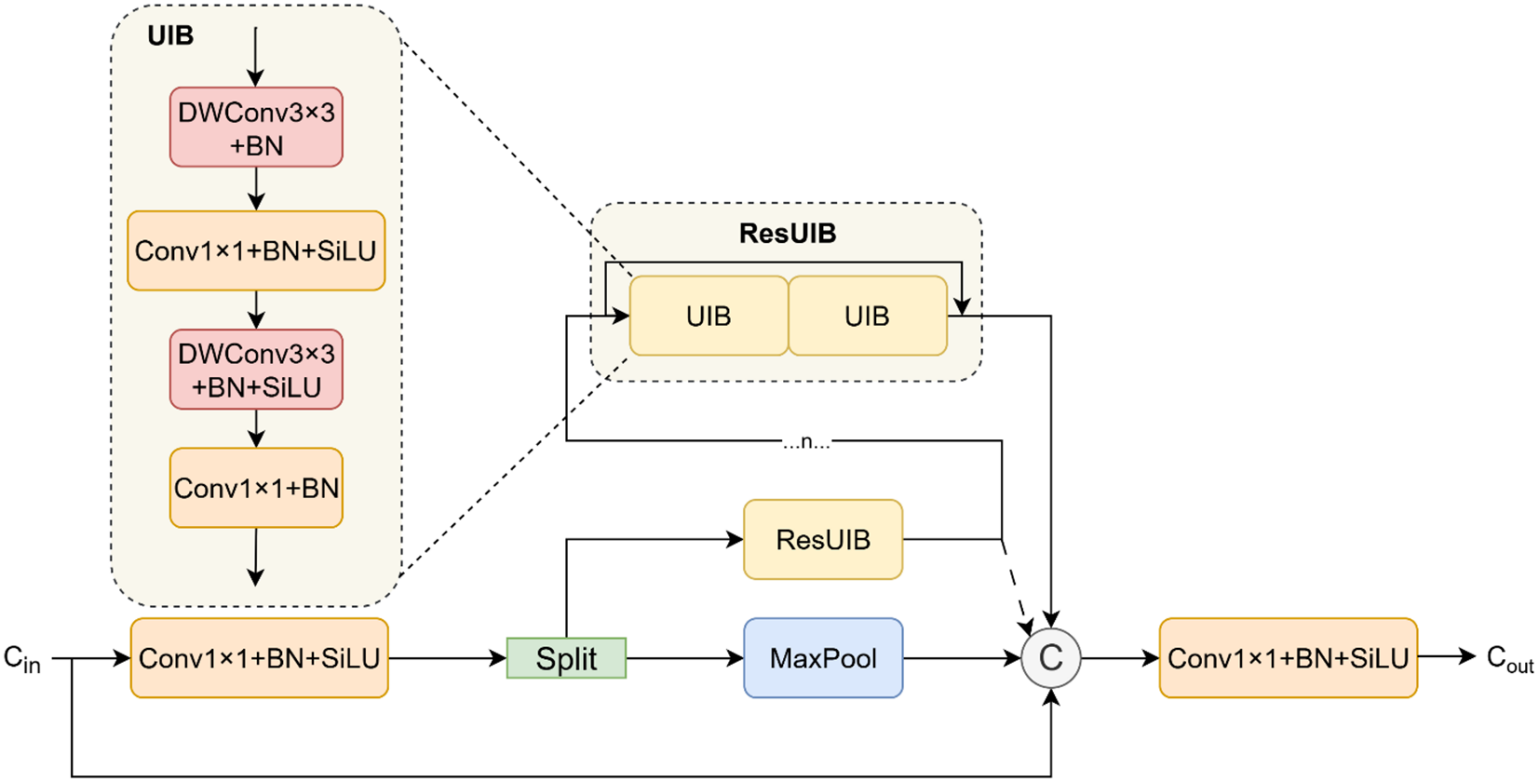
Structural diagram of the CSP-MUIB module, where e represents the scaling factor and E represents the expansion factor.

The C3K2 module (Khanam and Hussain, 2024b) essentially switches between the C3 (Khanam and Hussain, 2024a) and C2F modules (Yaseen, 2024) for different scenarios. To maintain high feature extraction capabilities while reducing computational complexity, we thoroughly analyzed the distinct characteristics of the C3 and C2F modules. While the C3 module optimizes computational efficiency via cross-stage partial connections, its feature fusion capability is limited. The C2F module, conversely, significantly enhances feature representation through gradient-dense connections, but at a considerable computational cost. Motivated by this inherent trade-off in existing backbone networks, we designed and proposed the novel Cross-Stage Pooling Unified Inverted Block (CSP-MUIB). As illustrated in **Figure 5**, it aims to achieve more powerful multi-granularity feature fusion at a lower computational cost. This module adheres to the Cross-Stage Partial (CSP) design philosophy. The input feature tensor is first expanded channel-wise via a 1×1 convolution and then split into two parallel paths. The first path employs a 3×3 max-pooling layer to efficiently extract local salient features while preserving spatial resolution. Meanwhile, the second path directs the remaining features into a stack of n Residual Unified Inverted Blocks (ResUIB) (Qin et al., 2024) for deep feature transformation. Each ResUIB consists of two residually connected Unified Inverted Blocks (UIB), which are derived from the advanced design of MobileNetV4. These UIBs adopt a computationally efficient inverted residual structure (a “Narrow-Expand-Narrow” channel pattern) and uniquely integrate multiple computational paradigms into a single block. Crucially, they can fuse convolutional and attention mechanisms to simultaneously capture local texture details and global structural dependencies. The CSP-MUIB module employs a dense feature aggregation strategy, concatenating the output from the max-pooling path, all intermediate features from the UIB stack (including the initial split features), and the original features retained through the cross-stage connection. This multi-level concatenation achieves a triple-complementary fusion of local, global, and cross-stage information. A final 1×1 convolution then fuses these rich, multi-receptive-field features and projects them to the target output channels. Through this meticulous design, the CSP-MUIB module effectively integrates local salient information, progressive deep features, and the original context. Consequently, it significantly enhances the model’s feature representation capabilities while maintaining computational efficiency, providing a superior feature foundation for downstream visual tasks.

#### 2.2.2 Progressive feature aggregator and deep feature synthesizer

To enhance feature representation across multiple scales within the feature pyramid—specifically, to enrich the semantic context of high-resolution feature maps and restore fine-grained details to low-resolution ones—we have designed two novel feature fusion modules: the Progressive Feature Aggregator (PFA) and the Deep Feature Synthesizer (DFS). These modules are engineered to effectively integrate crucial information from both shallow and deep layers of the network.

**Figure 6:**
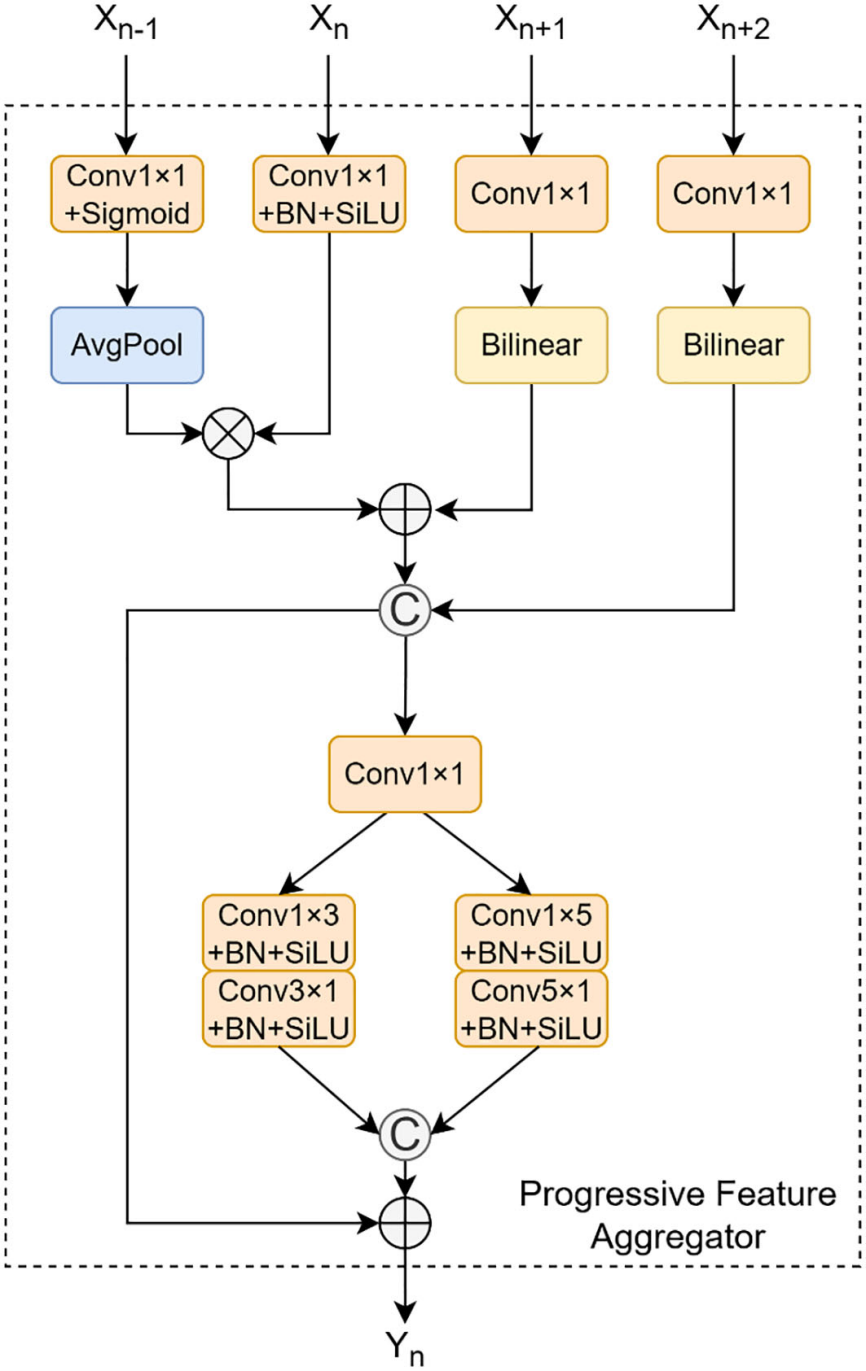
Structural diagram of PFA.


As illustrated in **Figure 6**, the PFA module is the first of our novel feature fusion units designed for the neck network of CPD-WeedNet. It aims to replace the simple element-wise addition or concatenation operations found in traditional Feature Pyramid Networks (FPNs), thereby enabling more efficient and refined multi-scale feature interaction. The PFA module specifically focuses on fusing features from levels P2 through P5 of the backbone and aligning the output to the spatial resolution of P3. Its core objective is twofold: to integrate high-resolution details from shallow layers with the rich semantic context from deep layers, and simultaneously, to introduce a lightweight spatial feature enhancement mechanism to improve the model’s representational capacity for small-to-medium-sized targets and complex textures. The module consists of three main stages: multi-scale feature pre-processing, feature fusion, and dual-branch asymmetric convolutional enhancement. In the pre-processing stage, the module takes the feature maps $F_{2},F_{3},F_{4},F_{5}$ from the P2 to P5 levels of the backbone as input, aligning them to the P3 spatial resolution $\left(H_{3},W_{3}\right)$ . Specifically, the input features are first adjusted channel-wise via 1×1 convolutions, while average pooling and bilinear interpolation are used for the necessary downsampling and upsampling to unify their spatial dimensions, resulting in the pre-processed features $\left(F_{2}^{,},F_{3}^{,},F_{4}^{,},F_{5}^{,}\right)$ . Subsequently, a preliminary fusion is performed through a strategy that combines element-wise addition, multiplication, and channel concatenation:

$$F_{inital}=Conv_{1\times 1}(Concat((F_{2}^{,}⊗F_{3}^{,})⊕F_{4}^{,}),F_{5}^{,})$$

To integrate information from different levels. In order to enhance the expression ability of spatial features without significantly increasing the computational cost, the PFA introduces a dual-branch asymmetric convolution structure. The initially fused feature $F_{inital}$ is fed into two parallel branches, namely $AC_{3}$ and $AC_{5}$ . The specific operations are shown in the following formulas:


$$AC_{3}(F_{inital})=SiLU(BN(Conv_{3\times 1}(SiLU(BN(Conv_{1\times 3}(F_{inital}))))))$$

$$AC_{5}(F_{inital})=SiLU(BN(Conv_{5\times 1}(SiLU(BN(Conv_{1\times 5}(F_{inital}))))))$$

The outputs of the two branches are then concatenated along the channel dimension and combined with $F_{inital}$ via a residual connection to generate the final output, $F_{PFA}$ . This progressive fusion and enhancement mechanism allows the PFA module to effectively facilitate cross-scale information interaction and improve the model’s perception of small-to-medium-sized targets and fine textures, all while maintaining computational efficiency.

**Figure 7:**
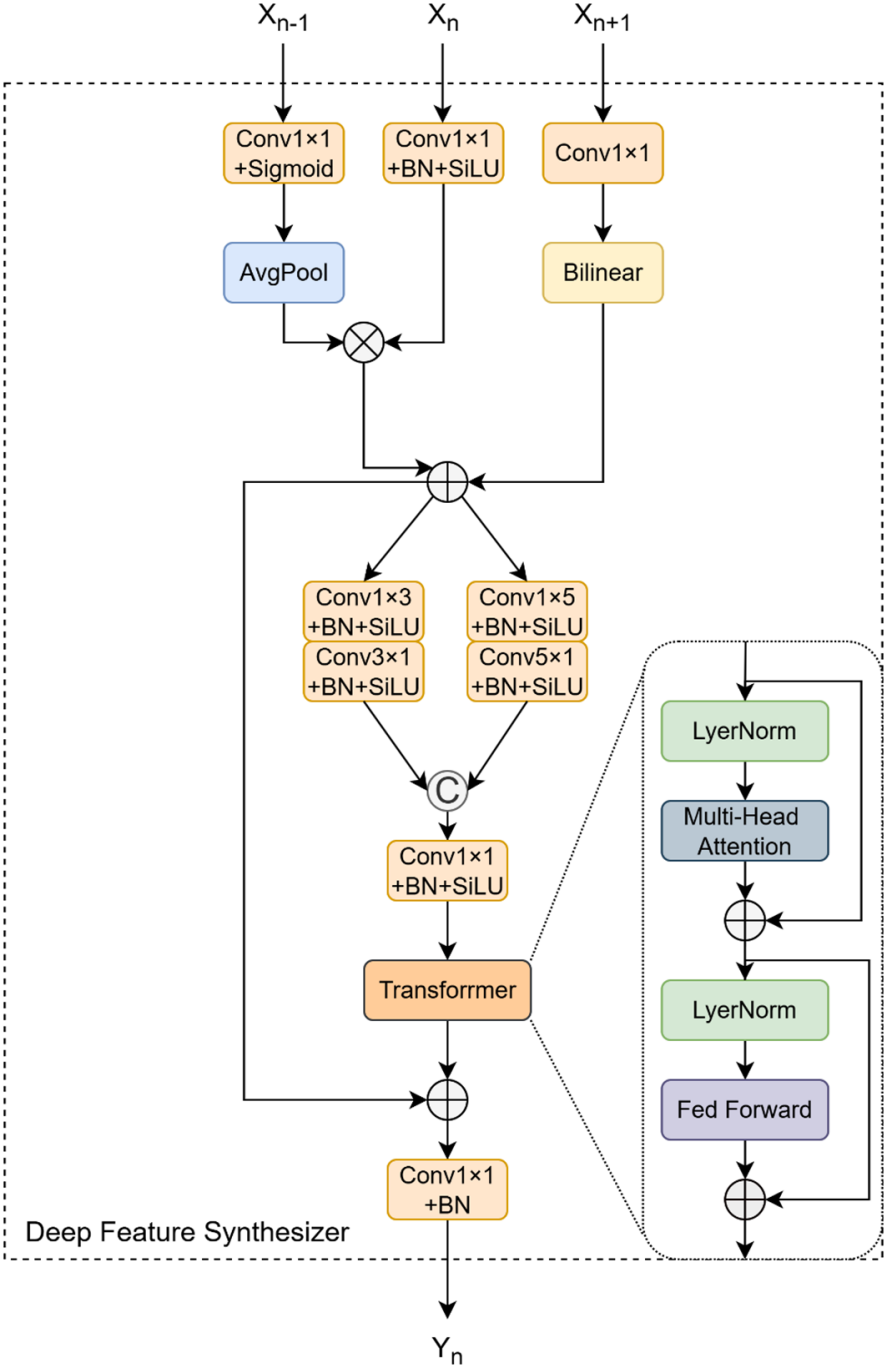
Structural diagram of DFS.


As shown in **Figure 7**, building upon the PFA module, we introduce the Deep Feature Synthesizer (DFS) module. The DFS module is responsible for integrating deeper-level features $\left(F_{3},F_{4},F_{5}\right)$ , aligning its output with the spatial resolution $\left(H_{4},W_{4}\right)$ of the P4 feature map, and adjusting the channel count to $C^{,,}$ . Initially, the module performs a pre-processing step on the input features, similar to that in the PFA, to obtain the normalized features $\left(F_{3}^{,,},F_{4}^{,,},F_{5}^{,,}\right)$ . These are subsequently combined using a preliminary fusion strategy that emphasizes deep-level features, expressed as follows:

$$F_{initial}^{,}=(F_{3}^{,,}⊗F_{4}^{,,})⊕F_{5}^{,,}$$

where ⊗ and ⊕ represent element-wise multiplication and addition, respectively. The resulting feature map, $F_{inital}$ is then fed into a dual-branch asymmetric convolutional structure, similar to that of the PFA module, for local feature refinement. This step generates two feature maps, $AC_{3}^{,}$ and $AC_{5}^{,}$ . The key innovation of the DFS module lies in the subsequent introduction of a Transformer block. The refined feature maps, $AC_{3}^{,}$ and $AC_{5}^{,}$ , are first concatenated along the channel dimension. The combined feature map is then reshaped via a “Rearrange” operation into a sequence of patch embeddings, $T\in {\mathbb{R}}^{B\times N\times D}$ , where N is the number of patches and D is the feature dimension of each patch. This sequence is then processed by a standard Transformer encoder layer, which consists of two core sub-layers: a multi-head self-attention mechanism and a position-wise feed-forward network, both supplemented with residual connections and layer normalization. Initially, the input sequence T is passed through the multi-head self-attention mechanism to produce an attention-weighted representation that captures the correlations between different patches. The output of this mechanism is then added to the original input T via a residual connection to form an intermediate output, $T^{,}$ :


$$T^{,}=T+Concat(softmax(\frac{Q_{1}K_{1}^{T}}{\sqrt{d_{k}}})V_{1},\ldots ,softmax(\frac{Q_{i}K_{i}^{T}}{\sqrt{d_{k}}})V_{i})W^{o}$$

Where $Q_{i},K_{i},V_{i}\in {\mathbb{R}}^{B\times N\times d_{k}}$ are the linear projections of the normalized input T into query, key, and value, where $d_{k}=\frac{D}{h}$ . The outputs of all heads are concatenated and passed through a final linear projection $W^{o}\in {\mathbb{R}}^{D\times D}$ . Subsequently, the output of the multi-head attention goes through layer normalization, then undergoes a position-wise non-linear transformation via the Feed-Forward Network (FFN), and is connected in a residual manner with $T^{,}$ . Finally, the output representation of the encoder layer is expressed as


$$T_{attn}=T^{,}+FFN(LN(T^{,}))$$

This series of operations enables $T_{attn}$ to integrate context information from the entire sequence. Finally, through the Rearrange operation, $T_{attn}$ is restored to the spatial structure of the feature map $F_{transformer}^{,}\in {\mathbb{R}}^{B\times 2C^{,,}\times H_{4}\times W_{4}}$ . The final output is obtained by combining the global information captured by the Transformer with the preliminarily fused features through an output-channel residual connection, and its calculation formula is:


$$F_{DPS}=F_{transformer}^{,}⊕Conv_{1\times 1}(F_{initial}^{,})\in {\mathbb{R}}^{B\times C_{out}^{,,}\times H_{4}\times W_{4}}$$

Through this design that combines the local perception ability of convolution and the global modeling ability of the Transformer, the DFS module can generate more informative feature representations. These representations are more adaptable to complex agricultural scenarios where there are large target-scale variations and occlusions.

#### 2.3 Experimental setup

In this experiment, to ensure the fairness of the evaluation index results, the default training image size is set to 640×640 pixels. All models adopt their respective default hyperparameter configurations, and network parameters are not initialized using pre-trained weights. The hardware configuration and software environment we used are shown in **Table 3**.

**Table 3:** Experimental environment configuration.

Environment configuration	Parameter
Operating system	Ubuntu 22.04.3 LTS
CPU	Intel(R) Xeon(R) Gold 6148 CPU @ 2.40GHz
GPU	2*A100(80GB)
Development environment	PyCharm 2023.2.5
Language	Python 3.9.0
frame	PyTorch 2.0.1
Operating platform	CUDA 11.8

### 2.4 Evaluation indicators

To comprehensively evaluate our model’s performance and its deployment potential on resource-constrained platforms, our evaluation framework combines two dimensions: accuracy and efficiency. The accuracy assessment for YOLO-style segmentation models considers both instance-level performance, such as mean Average Precision (mAP), and pixel-level classification accuracy, such as mean Intersection over Union (mIoU).

The instance-level metrics mainly include Precision (P), Recall (R), and mean Average Precision (mAP). These metrics are calculated based on the matching results between the predicted masks and the ground truth when they exceed a preset Intersection over Union threshold. Precision measures the accuracy of predictions, with a high P indicating a low false-positive rate. Recall measures the completeness of detection, with a high R indicating a low false-negative rate. mAP comprehensively reflects the overall performance of the model in balancing precision and recall. We report mAP50 (with an IoU threshold of 0.5) and the more stringent mAP50:95 (the average mAP with IoU thresholds ranging from 0.5 to 0.95). The calculation formulas are as follows.


$$P_{Box/Mask}=\frac{TP}{\left(TP+FP\right)}$$

$$P_{Box/Mask}=\frac{TP}{\left(TP+FP\right)}$$

$$AP_{N_{cl}}(t)=\int_{0}^{1}P_{N_{cl}}(R|IoU\geq t)dR$$

$$mAP50=mAP(0.5)=\frac{1}{N_{cl}}\sum_{cl=1}^{N_{cl}}P_{N_{cl}}(0.5)$$

$$mAP50:95=\frac{1}{\left|T\right|}\sum_{t\in T}mAP(t)$$

where TP represents the correctly detected targets, FP represents the falsely detected targets, FN represents the undetected targets, $N_{cl}$ is the number of classes, and the set of thresholds T={0.50,0.55,…,0.90,0.95} with |T|=10 thresholds. The pixel-level metrics mainly include mean Pixel Accuracy (mAcc) and mean Intersection over Union (mIoU). mAcc measures the average proportion of pixels of all classes that are correctly classified. mIoU calculates the intersection over union of the predicted mask and the ground-truth mask for each class and then takes the average. It is the golden standard for evaluating the overall quality of the segmentation mask and the accuracy of the boundaries, and is relatively insensitive to the problem of class imbalance. The calculation formulas are as follows.


$$mAcc=\frac{1}{N_{cl}}*\sum (\frac{TP_{i}}{\left(TP_{i}+FN_{i}\right)})$$

$$mIoU=\frac{1}{N_{cl}}*\sum (\frac{TP_{i}}{\left(TP_{i}+FN_{i}+FP_{i}\right)})$$

Where $TP_{i}$ and $FN_{i}$ are the pixel-level true-positive and false-negative counts for the *i*-th class.

## 3 Results

### 3.1 Performance on the self-constructed dataset

**Figure 8:**
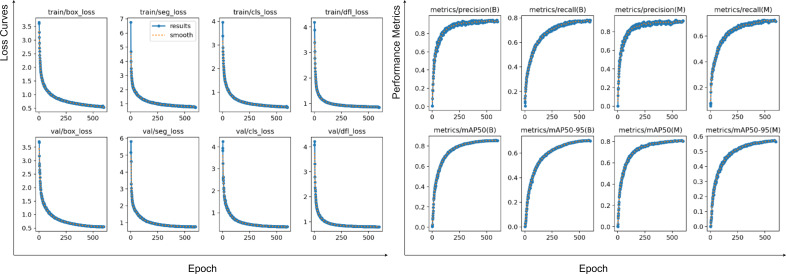
Changes in training and validation results and loss function of CPD-WeedNet on the self-constructed dataset.

In this experiment, the model was trained for 600 epochs with a batch size of 64 on 640×640 pixel images. As depicted in the accompanying **Figure 8**, the loss function curves (left four columns) for all terms—including ${\text{box}}_{\text{loss}}$ , ${\text{seg}}_{\text{loss}}$ , ${\text{cls}}_{\text{loss}}$ , and ${\text{dfl}}_{\text{loss}}$ —exhibited a rapid initial descent before stabilizing at approximately 350–550 epochs, indicating that the model effectively learned features from the training set. Crucially, the convergence trend of the validation loss closely mirrored that of the training loss, showing no significant rebounds or sustained increases. This demonstrates the model’s strong generalization capabilities and its success in avoiding overfitting or underfitting throughout the training process. Concurrently, the evaluation metric curves (right four columns) further corroborate these findings. On the validation set, both mAP50 and mAP50:95 metrics steadily improved with each epoch, eventually saturating near their optimal values in the later stages of training.

To comprehensively and quantitatively evaluate the performance of the proposed model, we conducted an exhaustive comparison between it and various state-of-the-art models under a unified experimental setup. In the evaluation on the self-constructed dataset, as shown in **Table 4**, our model demonstrated an overall leading edge in all key performance indicators, establishing a new best-in-class level. In terms of box detection, the model achieved an mAP50 of 85.3% and an mAP50:95 as high as 70.4%. Compared with the second-best performing YOLOv9c-seg, there were significant improvements of 2.0 and 3.0 percentage points, respectively. Meanwhile, its detection precision and recall rate reached 94.9% and 77.2% respectively, also the highest among all the compared models. Regarding mask segmentation, our model also demonstrated outstanding performance, with an mAP50 of 80.6% and an mAP50:95 of 56.9%. Compared with YOLOv9c-seg, the model with the best mAP50 (Mask) among the compared models, our model showed an improvement of 2.0 percentage points. In terms of the Mask mAP50:95 indicator, compared with the sub-optimal performance of YOLOv9c-seg, there was an improvement of 1.5 percentage points, and its P(Mask) and R(Mask) were also ahead of other models. Furthermore, in terms of overall segmentation accuracy, the model obtained an mIoU of 86.6% and an mAcc of 94.6%. These two core indicators outperformed all the compared models. Importantly, when achieving the above-mentioned comprehensively leading accuracy indicators, the model’s parameter count, computational load, and inference time were basically on a par with lightweight models such as YOLOv8s-seg and YOLO11s-seg. Even compared with YOLOv9c-seg, which has far more parameters and computational load than ours, our model not only surpassed it in all accuracy indicators but also reduced the parameter count by approximately 57%, decreased the computational load by approximately 73%, and had an inference speed 3.2 times that of YOLOv9c-seg. This fully demonstrates that our model has achieved a high-level unity and excellent balance between accuracy and efficiency on the self-constructed dataset.

**Table 4:** Performance comparison with other models on the self-constructed dataset.

Model	Box(%)				Mask(%)				mIoU(%)	mAcc(%)	Pare(M)	FLOPS(G)	Time(ms)
	P	R	mAP50	mAP50:95	P	R	mAP50	mAP50:95					
YOLOv8s-seg (Yaseen, 2024)	91.8	73.4	81.6	64.7	88.5	68.3	76.4	52.8	82.1	89.4	10.5	37.5	2.56
YOLOv9c-seg (Wang et al., 2024b)	92.4	75.0	83.3	67.4	88.8	70.5	78.6	55.4	84.6	92.7	23.5	138.3	8.41
YOLOv10s-seg (Wang et al., 2024a)	91.2	74.8	83	67.5	89.1	70.0	78.2	55.2	84.4	92.4	7.9	35.6	2.76
YOLOv11s-seg (Khanam and Hussain, 2024b)	91.9	77.2	82.4	66.7	87.8	70.2	77.6	54.7	83.3	91.4	10.1	35.4	2.73
Our model	94.9	77.2	85.3	70.4	89.4	73.3	80.6	56.9	86.6	94.6	10.1	37.8	2.64

### 3.2 Performance on the Fine24 dataset

On the large-scale multi-class Fine24 dataset, as shown in **Table 5**, our model also demonstrated strong competitiveness. In terms of box detection, its mAP50 reached 73.3%, outperforming all compared YOLO models; the mAP50:95 reached 51.9%, basically on a par with YOLO11s-seg. Regarding mask segmentation, the model also showed a leading performance in the mAP50 and mAP50:95 indicators, which were 65.9% and 37.4% respectively, representing an increase of 0.9-2.9 and 0.2-1.4 percentage points compared to other YOLO models. In terms of overall segmentation accuracy, the model achieved an mIoU of 75.4% and an mAcc of 81.7%. Compared with YOLOv8s-seg and YOLOv11s-seg, which have similar parameter counts, our model significantly improved the mIoU by 12.1 and 9.4 percentage points, respectively, and the mAcc by 11.8 and 9.6 percentage points, respectively. Even compared with YOLOv9c-seg, which has more parameters and computational load, our model still maintained significant advantages of 9.1 and 9.3 percentage points in mIoU and mAcc, respectively. Particularly, compared with the SOTA model SWFormer, which was also evaluated on the Fine24 dataset, our model had only a slight gap of 1.1 and 2.2 percentage points in mIoU and mAcc, respectively. However, this highly competitive accuracy was achieved with only about one-fifth of the parameter count of SWFormer, approximately one-fourteenth of its computational load, and a nearly 58-fold faster inference speed. This characteristic of trading acceptable accuracy for significant efficiency gains strongly demonstrates the excellent ability of our model design to balance high performance and extreme efficiency, making it particularly suitable for applications with limited resources and high real-time requirements.

**Table 5:** Performance comparison with other models on the Fine24 dataset.

Model	Box(%)				Mask(%)				mIoU(%)	mAcc(%)	Pare(M)	FLOPS(G)	Time(ms)
	P	R	mAP50	mAP50:95	P	R	mAP50	mAP50:95					
YOLOv8s-seg (Yaseen, 2024)	73.8	63.8	68.9	50.1	72.3	57.3	62.1	36.0	63.3	69.9	10.5	37.5	2.27
YOLOv9c-seg (Wang et al., 2024b)	75.4	66.9	71.9	51.7	72.3	59.4	65.0	37.2	66.3	72.4	23.5	138.3	7.28
YOLOv10s-seg (Wang et al., 2024a)	75.0	66.8	71.0	51.8	72.8	59.8	63.8	37.0	65.4	73.0	7.9	35.6	2.42
YOLOv11s-seg (Khanam and Hussain, 2024b)	76.6	66.1	71.2	52.0	70.6	60.0	63.0	36.7	66.0	72.1	10.1	35.4	2.49
SWFormer (Jiang et al., 2024)	–	–	–	–	–	–	–	–	76.5	83.9	52.3	527.5	137
Our model	76.9	67.1	73.3	51.9	73.2	62.0	65.9	37.4	75.4	81.7	10.1	37.8	2.38

Here, “-” indicates that the results of these indicators were not mentioned in the corresponding model papers.

**Figure 9:**
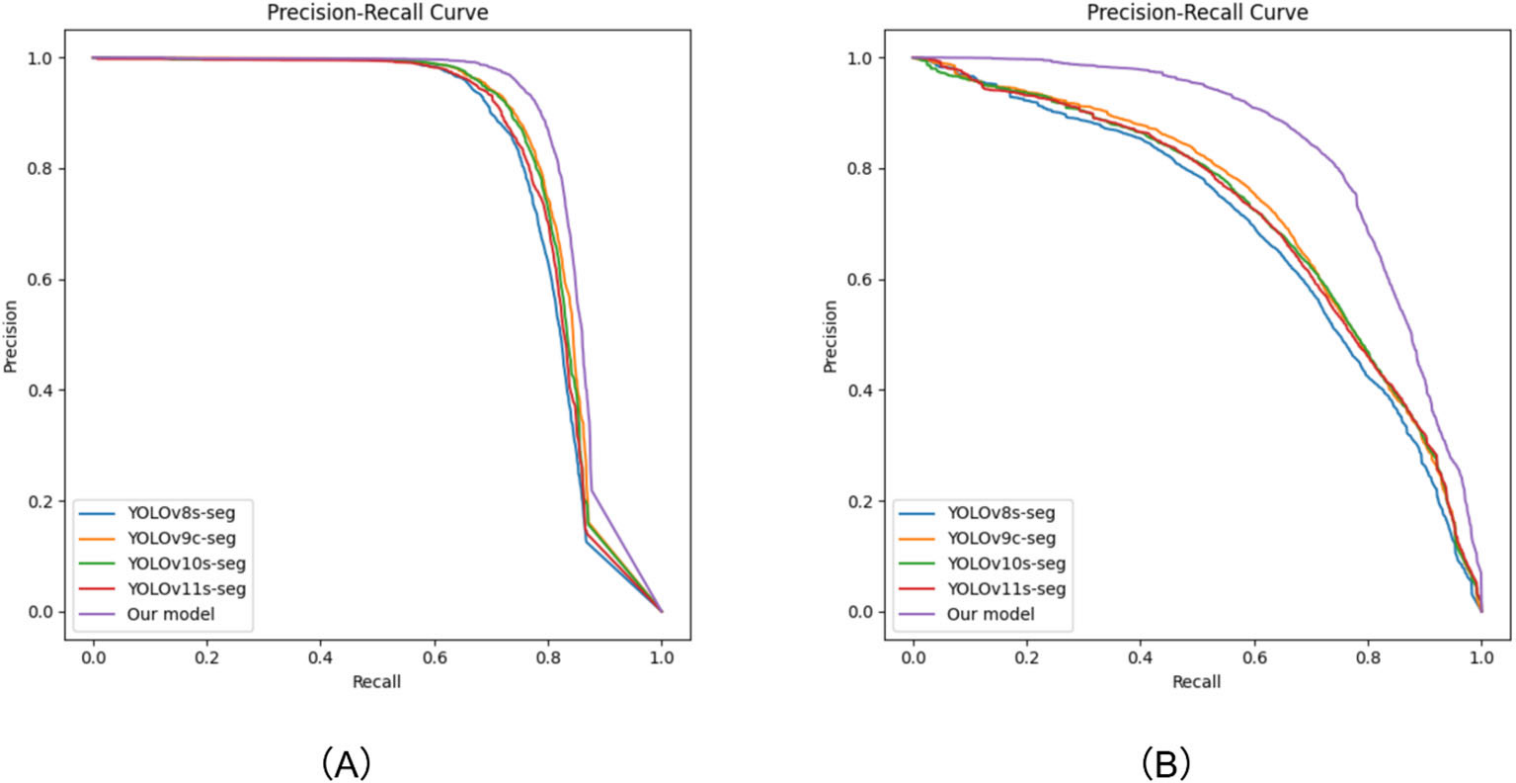
Precision-Recall curve graphs. **(A)** Obtained from the prediction on the test set of the self-constructed dataset; **(B)** Obtained from the prediction on the test set of the Fine24 dataset.

To objectively evaluate the detection and segmentation performance of the proposed model, this paper compares its precision-recall curve with that of the YOLO baseline model, as shown in **Figure 9**. The curve graph clearly shows that compared with the baseline model, the PR curve of our model not only covers a larger area, but also is generally located in the upper-right region of the other curves. This indicates that while our model can identify and segment as many targets as possible, it can also effectively control false positives. This characteristic is highly consistent with the stringent requirements for precise identification in the weeding application scenario.

Judging from the above experimental data, the excellent balance achieved by our model among high precision, low resource consumption, and high inference speed fully demonstrates that the model holds great potential and application value in scenarios with high demands for both efficiency and performance, such as real-time weeding systems for drones and automated lawn-maintenance robots.

### 3.3 Ablation studies

To systematically evaluate the effectiveness of the proposed modules, we conducted an ablation study using YOLOv11-seg as the baseline. We gradually introduced the CSP-MUIB, PFA, and DFS modules and evaluated their performance under the same experimental settings. Each improvement method was added sequentially. To ensure fairness and impartiality of the experiments, all experiments were carried out under consistent conditions. The experimental results are presented in **Table 6**.

**Table 6:** Results of the ablation experiment.

Model	Box(%)				Mask(%)				Pare(M)	FLOPS(G)
	P	R	mAP50	mAP50:95	P	R	mAP50	mAP50:95		
base	91.9	77.2	82.4	66.7	87.8	70.2	77.6	54.7	10.1	35.4
+CSP_MUIB	92.9	76.9	84.8	68.8	90.3	70.6	79.4	55.4	9.7	34.8
+ PFA	94.0	77.5	85.3	69.9	88.9	72.3	79.5	56.2	10.0	36.4
+DFS	91.7	76.6	84.2	68.1	90.4	70.5	79.1	55.1	9.9	34.1
+CSP_MUIB+PFA	94.2	77.7	85.3	69.9	91.0	71.2	80.0	56.1	10.0	37.2
+CSP_MUIB+PFA+DFS	94.9	77.2	85.3	70.4	89.4	73.3	80.6	56.9	10.1	37.8

#### 3.3.1 Performance analysis of the CSP-MUIB module on feature extraction in the segmentation network

**Figure 10:**
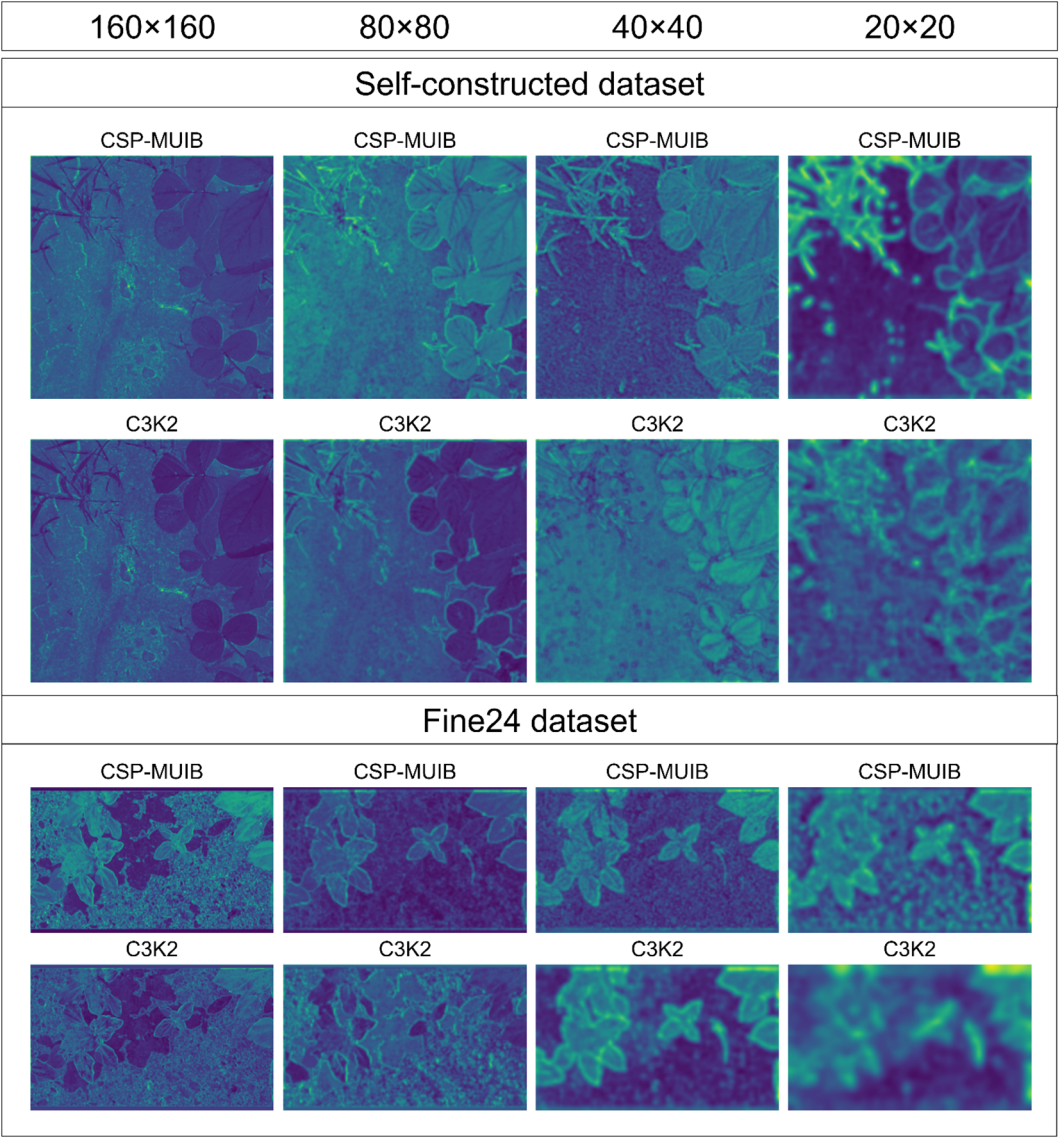
Feature map visualization comparing the proposed CSP-MUIB module with the baseline C3K2 module at decreasing spatial resolutions (160x160 down to 20x20) within the backbone. The results illustrate CSP-MUIB’s enhanced feature extraction capability, characterized by sharper focus on plant leaves and better suppression of background noise, maintaining more discriminative information even at lower resolutions compared to C3K2.

To verify the contribution of the CSP-MUIB module to the model’s feature extraction ability, we replaced the C3K2 module in the backbone network of the baseline model with the CSP-MUIB module, which is designed to enhance multi-granularity feature fusion. As shown in **Table 6**, this improvement led to significant performance enhancements. The mAP50 (Box) and mAP50 (Mask) increased by 2.4 and 1.8 percentage points, respectively, reaching 84.8% and 79.4%. To further confirm the effectiveness of CSP-MUIB in enhancing feature representation from a mechanistic perspective, we compared the feature maps output by it and the C3K2 module at different stages of the backbone network. As shown in **Figure 10**, by observing the feature visualization results from a resolution of 160×160 to 20×20, it can be clearly seen that compared with C3K2, the feature maps generated by CSP-MUIB can more sharply focus on the contours and key structural regions of plant leaves, while having less activation response to the background area. Even at the deeper stages of 40×40 and 20×20 with reduced resolution and more abstract features, CSP-MUIB can still better maintain the spatial information and distinctiveness of the targets. This visually superior feature quality intuitively explains why CSP-MUIB can improve the model’s segmentation and detection accuracy, preliminarily confirming its advantage in achieving stronger and more effective basic feature extraction at a relatively low computational cost.

#### 3.3.2 Performance analysis of PFA and DFS modules in multi-scale feature fusion for the segmentation network

**Figure 11:**
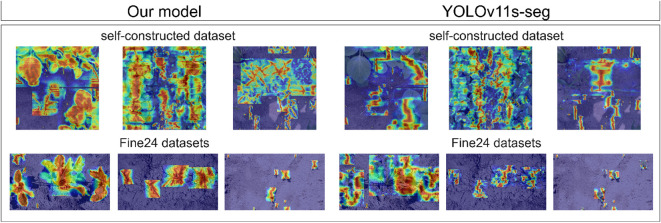
Qualitative comparison of neck network feature attention heatmaps between our proposed model and the baseline YOLOv11s-seg on six diverse image samples. The brighter regions indicate higher activation intensities. The heatmap of CPD-WeedNet covers the contours of target plants more accurately and completely, suggesting that its feature fusion module can focus more effectively on the entire target.

To verify the contributions of the PFA and DFS modules to enhancing the feature fusion ability, we conducted an ablation study. In the neck network, PFA enhances spatial details through multi-scale interactions and asymmetric convolutions, while DFS combines CNN and Transformer to capture global context. The quantitative results, as shown in **Table 6**, indicate that the performance of CPD-WeedNet, which integrates these two modules, has significantly improved. The mAP50 (Box/Mask) reached 85.3%/80.6%, and the mAP50:95 (Box/Mask) reached 70.4%/56.9%. Although DFS increases the computational cost, due to the efficiency optimization of PFA, the final increase in model parameters and computational load compared to the baseline is minimal, thus balancing performance and efficiency. The comparison of feature heatmaps in **Figure 11** shows that our model can precisely focus on the complete plant contours, while the baseline model’s focus is more dispersed. Through comprehensive quantitative and qualitative analyses, it is proven that PFA and DFS effectively enhance the feature fusion ability. By optimizing multi-scale interactions, spatial representation, and global context, they improve the detection and segmentation accuracy, especially demonstrating better performance in outlining the complete target contours.

### 3.4 Visualization of results

#### 3.4.1 Visualization of results on the self-constructed dataset

**Figure 12:**
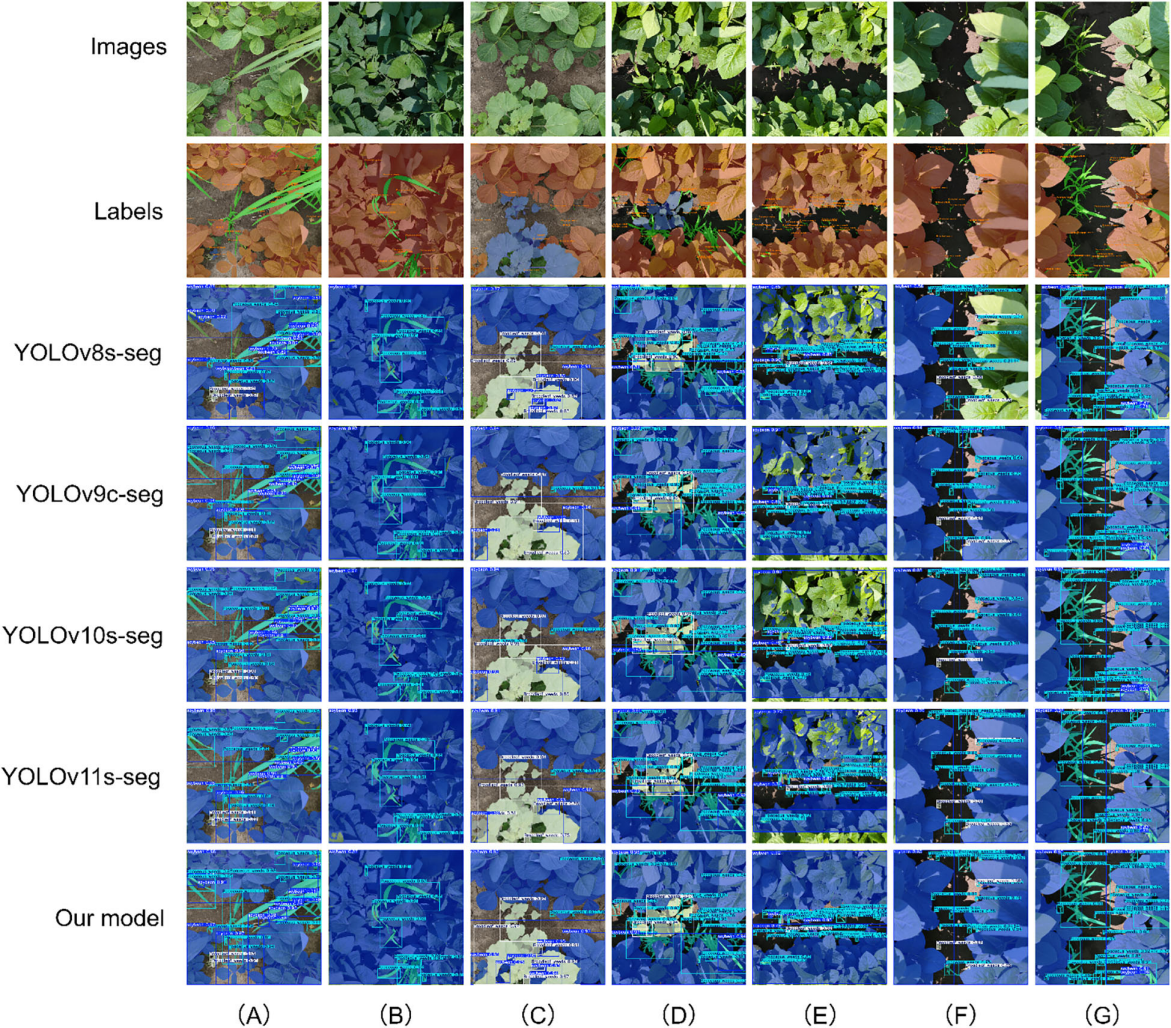
Qualitative comparison of segmentation results on the self-constructed dataset, highlighting model performance under various challenging scenarios. The rows, from top to bottom, display the original image, the ground-truth label, and the prediction results from different models. The columns illustrate performance under specific challenges: **(A)** severe occlusion of soybean by poaceous weeds; **(B)** overlapping poaceous weed targets; **(C)** severe occlusion of soybean by broadleaf weeds; **(D)** a small object detection scenario; **(E)** interference from strong light reflection; **(F)** misclassification of visually similar classes; **(G)** incomplete segmentation caused by partial occlusion.

To intuitively assess the recognition and segmentation accuracy of the CPD-WeedNet model proposed in this study for soybeans, poaceous weeds, and broadleaf weeds with varying degrees of overlap in complex field environments, we selected seven representative images (**Figures 12A–G**) from the test set for qualitative analysis and comparison. In scenarios with severe occlusion, such as in **Figure 12A** where the soybean leaves are heavily covered by poaceous weeds, and in **Figure 12C** where the soybean leaves at the bottom are blocked by broadleaf weeds, compared to other competing models, only the proposed model can successfully and accurately segment the occluded soybean leaf regions. Moreover, for the soybean target in the lower-left corner of **Figure 12C**, the proposed model does not suffer from the common issues of missed detection or misclassification that occur in other models. For the case of overlapping poaceous weeds targets shown in **Figure 12B**, the segmentation mask generated by the proposed model can fully cover the targets, effectively preventing under-segmentation or missed-segmentation problems caused by overlapping.

In **Figures 12D–G**, which contain various challenging factors, the proposed model demonstrates the overall best performance in terms of localization accuracy and segmentation quality. Specifically, in **Figure 12D**, although models such as YOLOv8s-seg, YOLOv10s-seg, and YOLOv11s-seg show poor pixel-level classification accuracy when segmenting soybeans, both the proposed model and YOLOv9c-seg can detect and segment the small-sized poaceous weeds located in the lower-left corner of the image. Moreover, the proposed model has a significantly higher detection confidence for this target. Facing the challenging situation of leaf surface reflection under strong sunlight in **Figure 12E**, most of the comparative models failed to identify a large area of soybean pixels because they could not extract features under such lighting conditions. However, thanks to the synergistic effect of the three innovative modules, CSP-MUIB, PFA, and DFS, integrated in the proposed model, it effectively overcomes the interference of strong light reflection and achieves accurate segmentation. Furthermore, under the more complex conditions shown in **Figure 12F** and **Figure 12G**, the comparative models expose more deficiencies. In **Figure 12F**, YOLOv8s-seg and YOLOv9c-seg not only miss the soybean area in pixel-level segmentation but also misclassify broadleaf weeds as poaceous weeds. In **Figure 12G**, when facing the occlusion of other targets by poaceous weeds on the right, the comparative models generally suffer from missed detection and incomplete pixel classification. For these complex situations, the proposed model achieves precise localization and segmentation of all targets, especially excelling in handling soybeans occluded by weeds and small-sized weeds occluded by soybeans.

In summary, the above-mentioned visualization results strongly demonstrate the robustness and superiority of the proposed model under variable lighting conditions and in complex field scenarios such as the mixture of soybeans and multiple types of weeds, as well as severe occlusion. The model can effectively overcome the common recognition challenges faced by traditional methods under these circumstances, achieving instance segmentation with higher accuracy.

#### 3.4.2 Visualization of Results on the Fine24 Dataset

To visually showcase the recognition performance of CPD-WeedNet compared to the baseline model for multi-category targets of different scales, and to intuitively verify the comprehensive effects of the introduced modules, we selected a series of representative images for visual analysis. These images encompass targets of various sizes and quantities, as well as diverse levels of complexity in field scenarios.

**Figure 13:**
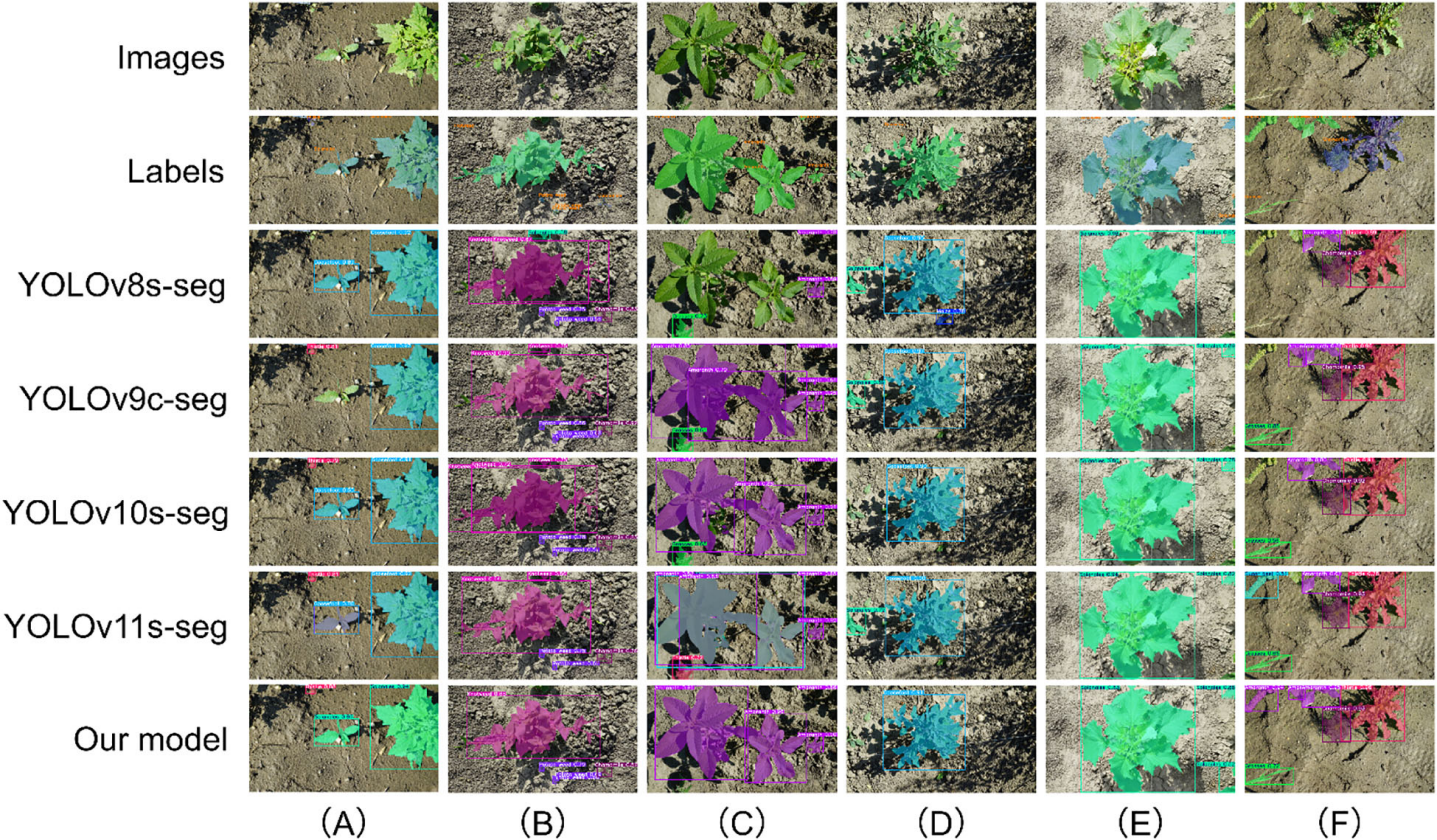
Qualitative results for large-sized targets on the Fine24 dataset. The rows display the original image, ground-truth label, and predictions from various models. The columns highlight performance in specific scenarios: **(A)** precise segmentation of a target with distinct contours; **(B–E)** robust performance in cases involving complex backgrounds and partial occlusion; **(F)** superior recall in a scenario prone to missed detections by competing models.

**Figure 13** presents the performance of various models in the task of segmenting large-sized targets. In such scenarios, CPD-WeedNet demonstrates remarkable and robust performance. For instance, in **Figure 13F**, compared with the prevalent missed-detection issue among the comparative models, CPD-WeedNet significantly enhances the detection recall rate. Meanwhile, in the complex backgrounds or when there is partial occlusion between targets as depicted in **Figures 13B–E**, CPD-WeedNet effectively reduces misclassification and false-alarm occurrences. Notably, as shown in **Figure 13A**, for large-sized targets with distinct contours, CPD-WeedNet is capable of generating more elaborate and accurate segmentation masks.

**Figure 14:**
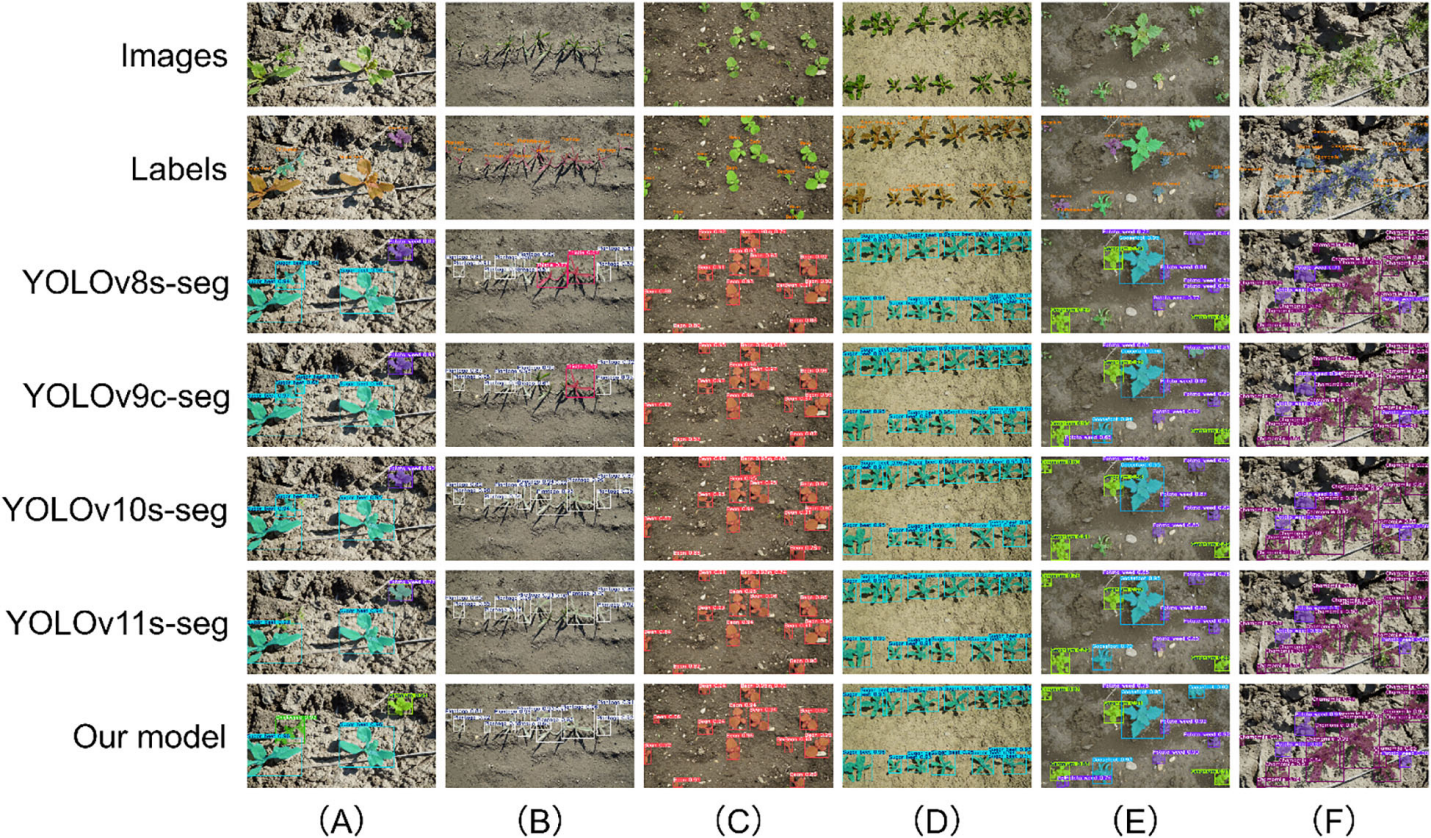
Qualitative segmentation results for medium-sized targets on the Fine24 dataset. The rows display the original image, ground-truth label, and predictions from various models. The columns highlight the model's robustness in multi-target scenarios: **(A)** high-precision segmentation, avoiding errors common in baseline models; **(B, E)** reduction of misclassification in dense scenes; **(C, D)** consistent detection in multi-target environments; **(E)** accurately distinguishing closely arranged targets; **(F)** maintaining precise contours for partially occluded targets.

**Figure 14** focuses on the segmentation effects of medium-sized targets. In the scenario shown in **Figure 14A**, CPD-WeedNet achieves high-precision target recognition and segmentation, effectively avoiding the common missed-detection and misclassification problems in the baseline models. When the number of targets increases and occlusion or proximity occurs, CPD-WeedNet demonstrates greater robustness. Specifically, it significantly reduces the frequently occurring missed-detection phenomenon in the baseline models (as shown in **Figures 14B–F**) and decreases misclassification of categories (as shown in **Figure 14B** and **Figure 14E**). Even under challenging conditions such as distinguishing closely arranged targets (as in **Figure 14E**) and providing accurate contours for partially occluded targets (as in **Figure 14F**), CPD-WeedNet still exhibits excellent performance.

**Figure 15:**
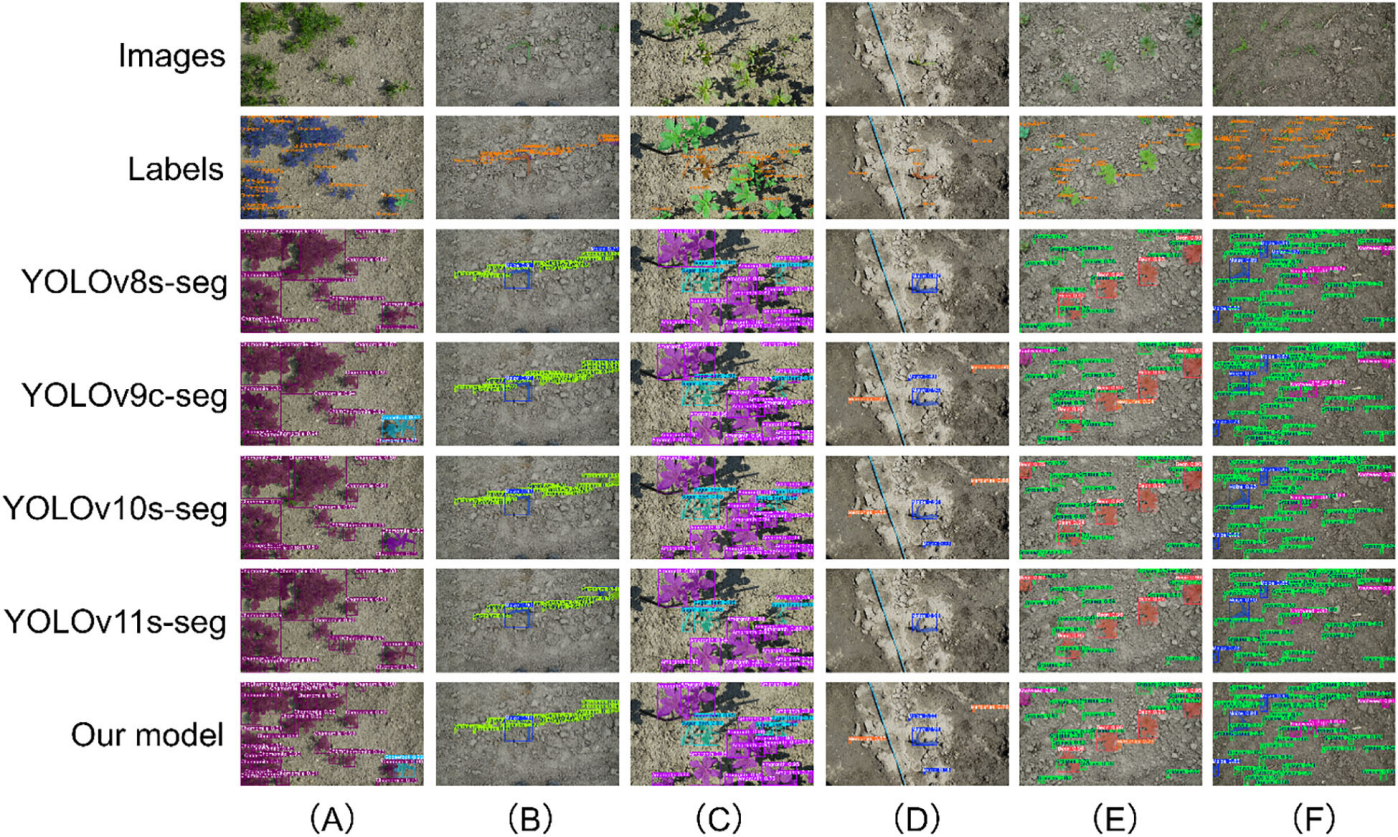
Qualitative segmentation results for small-sized targets on the Fine24 dataset, demonstrating model superiority in extremely complex scenarios. The rows compare model predictions against the ground-truth. The columns are organized to showcase three core strengths: **(A)** a superior ability to distinguish highly adjacent and overlapping targets; **(B–E)** exceptional detection integrity in complex scenes, including dense clusters **(B, C)** and cluttered backgrounds containing very small targets **(D, E)**; and **(F)** high-fidelity segmentation of partially occluded targets, a scenario where competing models often fail.

For small-sized targets, the performance advantage of CPD-WeedNet is particularly prominent. **Figure 15** shows the segmentation results of each model in extremely complex scenarios with a large number of dense and significantly overlapping small-sized targets. Under such conditions, CPD-WeedNet once again proves its superiority: First, it achieves higher target detection integrity. For example, it exhibits the lowest missed-detection rate in **Figure 15B** and **Figure 15F**. Second, it has a better ability to distinguish highly adjacent or overlapping targets, as shown in **Figure 15A**. Third, its segmentation accuracy when dealing with partially occluded small targets is also higher, as shown in **Figure 15F**. It is worth noting that in challenging scenarios involving numerous small targets, such as the dense clusters in **Figures 15B**, **C**, the cluttered background with extremely small targets in **Figure 15D**, and the complex textured background in **Figure 15E**, only CPD-WeedNet can achieve accurate segmentation and localization of almost all targets, while other comparative models experience varying degrees of detection and segmentation failures.

In summary, the multi-category visualization results across different target sizes, quantities, and scene complexities consistently indicate that CPD-WeedNet consistently and significantly outperforms the baseline models in terms of reducing detection errors, enhancing classification accuracy, and improving segmentation quality. These qualitative analysis results strongly confirm the effectiveness of the proposed improvement strategies, as well as the overall robustness and generalization of the CPD-WeedNet model.

## 4 Discussion

In this study, we successfully constructed and validated CPD-WeedNet, a lightweight framework designed to address the core challenges of fine-grained weed segmentation in precision agriculture. Our key contribution lies in achieving an excellent balance between computational efficiency and segmentation accuracy through the synergistic optimization of the backbone and neck networks. This was demonstrated by our experimental results. On our self-constructed dataset, our model achieved 80.6% mAP50 (Mask), significantly outperforming baselines like YOLOv8s-seg. More importantly, on the challenging Fine24 dataset, CPD-WeedNet demonstrated its competitive edge. Compared to larger models such as YOLOv9c-seg (66.3% mIoU) and the state-of-the-art SWFormer (76.5% mIoU), our model achieved a highly competitive 75.4% mIoU with only a fraction of the computational resources—approximately one-fifth of the parameters and one-fourteenth of the computational load of SWFormer. This result offers a compelling path forward in the prevalent dilemma, where high-performance models are often too cumbersome for practical deployment, while lightweight models sacrifice critical accuracy in complex scenarios. By proving the feasibility of balancing these two aspects, our work lays a solid algorithmic foundation for the future deployment of intelligent weeding systems on resource-constrained platforms.

Furthermore, situating our work within the broader context of machine learning research helps to highlight the universality of its methodological principles. The architectural design principles we employed to solve weed segmentation resonate with those in other fields that also require extracting fine-grained features from complex and noisy data, such as bioinformatics and medical image analysis. Although the specific problems in these domains differ, they share a core challenge: designing computational models that are both sensitive to subtle signals and robust to confounding information. For instance, Chen et al. employed a feature reconstruction technique to enhance biomarker discriminability in CBCT images (Chen et al., 2025). This aligns closely with the design philosophy of our CSP-MUIB module, as both approaches suggest a shared design principle: when the signal-to-noise ratio of the raw input is low, incorporating an explicit, multi-path feature refinement and enhancement stage in the backbone can be a more efficient strategy than relying solely on deeper network layers for “brute-force” learning. Similarly, the voting ensemble strategy used by Bao et al (Bao et al., 2023)and the synergistic mechanism of our PFA and DFS modules both embody the core idea of “enhancing robustness through information fusion.” The fundamental goal, whether fusing predictions from different models or, as in our case, feature maps from different scales and levels of abstraction, is to mitigate the limitations of a single information source, thereby making more reliable judgments when faced with uncertainties like ambiguity or occlusion. Furthermore, the attention mechanism has proven to be a powerful, cross-domain paradigm for dynamically allocating computational resources. Yuan et al. leverage attention modules to identify key genes in single-cell data analysis (Yuan et al., 2025b); similarly, the Transformer in our DFS module employs self-attention to capture long-range contextual relationships in images. This demonstrates that, regardless of data modality, enabling a model to preferentially allocate its finite computational resources to the most critical information has become a convergent trend in building high-performance deep models. While these advanced methodologies provide valuable conceptual blueprints, their successful application in precision agriculture cannot be achieved by simple transplantation, as each domain presents unique challenges. The core challenge in medical image analysis, for example, often involves distinguishing between morphologically highly similar tissues with subtle differences in standardized images. In contrast, the central challenge in precision agriculture stems from its open-world, unstructured environment, where models must be robust to the immense visual uncertainty introduced by drastic and uncontrollable illumination changes, severe occlusions, and complex backgrounds of soil and shadows. Therefore, our work is not a mere transplantation of these methodologies but a fruitful, innovative adaptation and optimization tailored to the unique nature of the agricultural scenario.

An in-depth analysis of the performance disparity between the self-constructed dataset (86.6% mIoU) and the Fine24 dataset (75.4% mIoU) reveals our model’s current strengths and limitations. This performance gap is primarily attributed to a sharp increase in task complexity and the challenge of domain shift. Our self-constructed dataset contains only three categories, whereas Fine24 comprises 24 fine-grained crop and weed classes, which not only elevates classification difficulty but also introduces significant challenges related to high inter-class similarity. This suggests that while our proposed CSP-MUIB backbone is highly efficient at feature extraction, its discriminative power may still have room for improvement in ultra-fine-grained tasks requiring the distinction of subtle textural differences. Consequently, we identify the model’s primary limitations as its discriminative capability for ultra-fine-grained classes and its generalization performance across unseen, disparate data domains.

To overcome the aforementioned limitations and further extend this research, we have charted several interconnected directions for future work. First, we will focus on enhancing the model’s discriminative power for ultra-fine-grained classes. In addition to exploring more advanced attention mechanisms, we plan to incorporate metric learning strategies to compel the model to learn a feature space that better separates morphologically similar species. Inspired by the work of Yuan et al (Yuan et al., 2024, Yuan et al., 2025a), we also envision using Graph Neural Networks (GNNs) to model the morphological relationships of weeds across different growth stages; by constructing a “growth-stage graph,” the model could potentially learn to leverage contextual information to aid recognition. Second, to improve the model’s generalization to new farm environments, we will integrate Unsupervised Domain Adaptation (UDA) techniques into our framework to train the model on learning domain-invariant features. Ultimately, as the critical step to verify our algorithm’s real-world value, we will deploy the optimized CPD-WeedNet model onto a physical robotic platform and integrate it with a control system to perform a complete, closed-loop validation of the autonomous operation, from visual perception to precise physical actuation.

## 5 Summary

In response to the challenges in farmland environments, such as overlapping and occlusion of weeds and crops under different lighting conditions, variable target scales, complex backgrounds, and limited computing resources, this study proposes and successfully validates a novel weed segmentation model-CPD-WeedNet. Based on in-depth optimization of YOLOv11-seg, this model significantly enhances its comprehensive performance through the innovative integration of three core components. First, the CSP-MUIB backbone module, with its unique structural design, not only effectively conserves computing resources but also enhances the network’s feature-encoding ability, generating highly discriminative initial feature representations for subsequent processing. Second, the PFA module deployed in the neck network focuses on mining and efficiently integrating high-resolution detail information from the shallow-layer network. Its progressive aggregation strategy and asymmetric convolution enhancement mechanism significantly improve the model’s ability to capture fine textures and accurately outline the contours of medium-and small-sized targets. Finally, the DFS module, also located in the neck, introduces the Transformer architecture, endowing the model with a powerful global scene context-understanding ability. By effectively combining the local perception advantages of convolution with the long-range dependence modeling characteristics of the self-attention mechanism, it is crucial for correctly interpreting large-sized targets, handling complex background interference, and resolving issues such as mutual occlusion among plants.

The test evaluation on the self-constructed dataset strongly demonstrates that, thanks to the synergistic effect of these three major modules, CPD-WeedNet significantly outperforms several mainstream YOLO baseline models in terms of segmentation accuracy and detection performance. For object detection (Box) and instance segmentation (Mask), it achieves an mAP50 of 85.3% and 80.6%, an mAP50:95 of 70.4% and 56.9% respectively. In the pixel-level segmentation task, it reaches an mIoU of 86.6% and an mAcc of 94.6%. Furthermore, the evaluation results on the public Fine24 dataset further highlight the excellent performance-efficiency balance of CPD-WeedNet. Compared with heavy-weight models participating in the evaluation of the same dataset, with only 10.1M parameters and a computational load of 37.8 GFLOPs, CPD-WeedNet achieves a highly competitive mIoU of 75.4% and an mAcc of 81.7% at an extremely fast inference speed of 2.38 ms. It is worth noting that its object detection and instance segmentation performance on this dataset are also excellent, reaching 73.3% mAP50 (Box) and 65.9% mAP50 (Mask) respectively. Qualitative visual analysis further corroborates the robustness and superiority of CPD-WeedNet in handling complex situations such as targets of different sizes, densities, occlusions, and blurred edges.

In summary, this study successfully developed and validated CPD-WeedNet, an efficient and accurate model specifically designed for fine-grained weed instance segmentation. This model provides critical visual perception support for the development of low-cost, real-time intelligent weeding systems. The core contribution of this research is the empirical demonstration that maintaining low computational load and high inference speed is feasible while achieving high accuracy. This excellent accuracy-efficiency balance underscores the model’s significant potential for deployment on resource-constrained platforms, such as UAVs and ground robots. Consequently, CPD-WeedNet holds broad practical application prospects for reducing reliance on chemical pesticides, enhancing agricultural production efficiency, and promoting sustainable agriculture.

## Data Availability

The self-constructed dataset presented in this study is not readily available as it is currently restricted due to its status as a core asset of an ongoing research project. Code for model training and evaluation on the public Fine24 dataset is available at https://github.com/DL019/CPD-WeedNet. For academic non-commercial purposes, limited access to the self-constructed dataset may be granted upon reasonable request to the corresponding authors, subject to a data use agreement. Requests to access the datasets should be directed to Lan Luo, 20231225@mails.jlau.edu.cn.
